# GSTM4 Suppresses Prostate Cancer Progression by Targeting TRIM27‐mediated ACLY Ubiquitination and Lipid Metabolism

**DOI:** 10.1002/advs.77394

**Published:** 2026-08-30

**Authors:** Kang‐Ping Xiong, Fei Liu, Wang Wang, Gang Wang, Kai‐Yu Qian, Sheng Tu, Si‐Ming Chen, Hui‐Min Xu, Shan‐Shan Zhang, Yong Li, Xing‐Huan Wang, Hong Weng

**Affiliations:** ^1^ Department of Medical Oncology The First Affiliated Hospital Jiangxi Medical College Nanchang University Nanchang China; ^2^ Department of Urology Hubei Key Laboratory of Urological Diseases Zhongnan Hospital of Wuhan University Wuhan China; ^3^ National Cancer Center/National Clinical Research Center for Cancer/Cancer Hospital Chinese Academy of Medical Sciences and Peking Union Medical College Beijing China; ^4^ Institute of Urology Wuhan University Wuhan China; ^5^ Department of Biological Repositories Human Genetic Resource Preservation Center of Hubei Province Zhongnan Hospital of Wuhan University Wuhan China; ^6^ Department of Obstetrics and Gynecology Ultrasound Zhongnan Hospital of Wuhan University Wuhan China; ^7^ Center For Evidence‐Based and Translational Medicine Zhongnan Hospital of Wuhan University Wuhan China; ^8^ Medical Research Institute Frontier Science Center for Immunology and Metabolism Taikang Center for Life and Medical Sciences Wuhan University Wuhan China; ^9^ Wuhan Research Center for Infectious Diseases and Cancer Chinese Academy of Medical Sciences Wuhan China

**Keywords:** ACLY, GSTM4, lipid synthesis, prostate cancer, TRIM27

## Abstract

Prostate cancer (PCa) is a prevalent malignancy in men, and lipid metabolic reprogramming contributes to its progression and therapeutic resistance. However, the drivers of lipid metabolic dysregulation in PCa remain incompletely defined. Through transcriptomic sequencing of PCa patient tissues, we identified glutathione S‐transferase mu 4 (GSTM4) as a novel regulator of lipid metabolism. GSTM4 was significantly downregulated in PCa tissues and negatively correlated with triglyceride content. Functionally, GSTM4 overexpression inhibited PCa cell proliferation and migration, thereby suppressing tumor development and bone metastasis. Mechanistically, GSTM4 acted independently of its classical detoxification activity by interacting with tripartite motif‐containing 27 (TRIM27) and ATP citrate lyase (ACLY), a key enzyme in de novo lipid synthesis. GSTM4 promoted TRIM27‐mediated ubiquitination and degradation of ACLY at lysine 554, reducing acetyl‐CoA production and lipid accumulation. This inhibition of lipid synthesis suppressed PCa growth and metastasis in vitro and in subcutaneous, orthotopic, and bone metastasis models. Moreover, ETC‐1002 (bempedoic acid), a clinically approved ACLY inhibitor, showed therapeutic potential against PCa. These findings reveal a non‐canonical GSTM4‐TRIM27‐ACLY axis that regulates lipid metabolism and represents a promising therapeutic target for advanced PCa.

## Graphical abstract

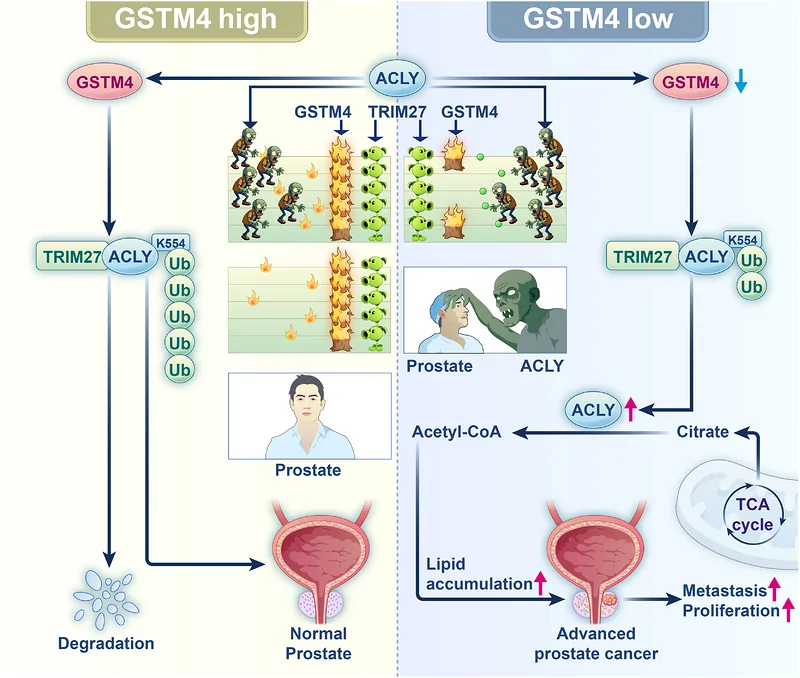

## 1 Introduction

Prostate cancer (PCa) remains a significant global health concern, ranking as the second most frequently diagnosed cancer and the fifth leading cause of cancer‐related deaths among men worldwide [1, 2]. In the United States, the incidence of PCa has increased by approximately 3% annually since 2014, making it the most commonly diagnosed cancer and the second leading cause of cancer death among American men [3]. While localized PCa has a favorable prognosis with a five‐year survival rate of up to 98%, the outlook drastically worsens upon metastasis, dropping survival rates to around 30% [4]. Bone metastases occur in approximately 70% to 90% of patients with hormone‐refractory PCa [5], and current systemic therapies offer only palliative benefits without significantly improving overall survival [6]. These statistics underscore an urgent need for novel therapeutic strategies that can effectively impede PCa progression and enhance patient outcomes.

Emerging evidence highlights the critical role of lipid metabolism in cancer development and progression [7]. Tumor cells reprogram lipid metabolic pathways to meet the demands of rapid growth and proliferation, engaging in enhanced de novo lipogenesis and fatty acid oxidation [8, 9, 10]. In PCa, the prostate gland is surrounded by adipose tissue, creating a lipid‐rich microenvironment that influences tumor behavior [11, 12, 13]. Recent studies have demonstrated that increased expression of fatty acid synthase (FASN) and accumulation of cholesteryl esters in PCa cells are closely linked to tumor aggressiveness and poor prognosis [14, 15, 16]. Key enzymes such as ATP citrate lyase (ACLY) and acetyl‐CoA carboxylase (ACC), pivotal in fatty acid synthesis, have been identified as potential therapeutic targets [17, 18, 19]. Moreover, lipid metabolic reprogramming has been implicated in therapy resistance, promoting survival under metabolic stress and facilitating metastatic dissemination [20, 21]. Despite these advances, the underlying molecular mechanisms driving lipid metabolism disorders in PCa remain incompletely understood. To elucidate the role of lipid metabolism in PCa, we performed RNA sequencing (RNA‐seq) analysis on patient‐derived PCa tissues and adjacent normal tissues. RNA‐seq analysis of selected samples revealed eight key genes associated with PCa and confirmed aberrant activation of 17 lipid metabolism–related pathways. Through correlation analysis and functional validation, we pinpointed glutathione S‐transferase mu 4 (GSTM4) as a pivotal regulator of lipid reprogramming in PCa.

The glutathione S‐transferase (GST) family comprises eight classes, differentiated by their amino acid sequences and substrate specificities [22]. GSTM4, belonging to the mu class, functions as a phase II detoxification enzyme, facilitating the conjugation of reduced glutathione with various electrophilic substrates, thereby contributing to cytoprotection [23]. Beyond detoxification, GSTs have roles in cellular signaling, post‐translational modifications, and chemotherapeutic drug resistance [24]. However, the role of GSTM4 in tumor biology remains contentious and underexplored. Our findings reveal that GSTM4 regulates PCa growth, migration, and lipid content independently of its traditional glutathione transferase activity. Notably, we observed a significant negative correlation between GSTM4 RNA levels and triglyceride content in PCa tissues. Mechanistically, GSTM4 forms a complex with tripartite motif‐containing 27 (TRIM27) and ACLY, enhancing TRIM27‐mediated ubiquitination and degradation of ACLY. This process inhibits de novo lipid synthesis and suppresses PCa progression. Our results suggest that targeting the GSTM4‐TRIM27‐ACLY axis could offer a promising therapeutic strategy for combating PCa.

## 2 Results

### 2.1 GSTM4 Was Downregulated in PCa and Correlated With Patient Prognosis and Lipid Metabolism

Abnormally active lipid metabolism and abundant lipid droplet content are hallmark features of PCa. To identify the specific pathways involved in lipid metabolism within the context of PCa, we performed pathway enrichment analysis using RNA‐Seq data from a cohort of PCa and adjacent non‐cancerous tissues collected by our research team (Figure 1A). Gene Set Enrichment Analysis (GSEA) revealed 17 lipid metabolism‐related pathways significantly enriched in PCa. Through comprehensive early‐stage screening, our team identified eight key genes potentially involved in PCa progression [25]. During the pre‐screening phase, we conducted a correlation analysis to explore the relationship between these eight genes and the 17 lipid metabolism pathways. This analysis demonstrated a significant association between all eight genes and lipid metabolism (Figure 1B). As shown in Figure 1B, among the eight candidate genes identified in our initial screening, seven genes (MCC, RAB9B, GSTM4, ASPA, RHBDL3, ANGPT1, and PI3KC2G) were negatively correlated with the indicated lipid metabolism pathways, whereas HOXC6 showed a positive correlation (Figure 1B). Following correlation ranking, we selected the top five genes for phenotypic validation. Overexpression models were established by transfecting PC‐3 and DU145 cells with corresponding plasmids (Vector, MCC, GSTM4, ASPA, RHBDL3, and RAB9B) (Figure S1A,B). Plate cloning assays and Transwell assays revealed that GSTM4 exerted the strongest inhibitory effect on the proliferation and migration of both PC‐3 (Figure 1C,D) and DU145 cells (Figure S2A,B). qRT‐PCR and RNA sequencing (RNA‐seq) analyses of PCa and adjacent tissues further confirmed a significant downregulation of GSTM4 in PCa (Figure 1E,F). Immunohistochemical staining of tissue microarrays also revealed markedly reduced GSTM4 protein levels in paired PCa samples (Figure 1G). In paired clinical samples, GSTM4 protein expression was consistently lower in tumor tissues compared to adjacent non‐cancerous tissues (Figure 1H,I and Figure S2C). To verify the association between GSTM4 and lipid metabolism, we analyzed GSTM4 RNA expression alongside corresponding triglyceride concentrations in PCa tissues. The results demonstrated a significant inverse correlation (Figure 1J). These findings were corroborated by data from The Cancer Genome Atlas (TCGA) (Figure 1K) and multiple GEO datasets (GSE88808, GSE32571, GSE62872, GSE70768) (Figure S2D), all of which confirmed consistent downregulation of GSTM4 RNA in PCa. Further examination of the TCGA database indicated a progressive decrease in GSTM4 RNA levels correlating with the advancement of PCa (Figure 1L and Figure S2E). Survival analysis utilizing data from TCGA indicated that PCa patients with low GSTM4 expression levels had a poorer prognosis (Figure 1M).

**FIGURE 1:**
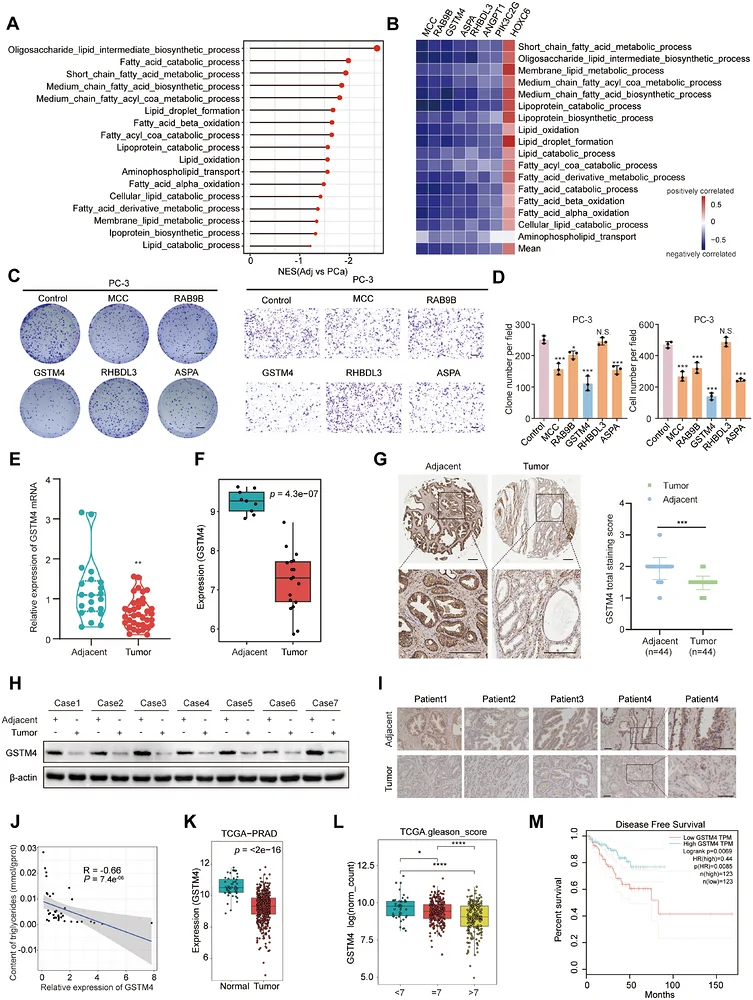
GSTM4 was downregulated in PCa and negatively correlated with patient prognosis and lipid metabolism. (A) Gene Ontology‐ Biological Process (GO‐BP) analyses of lipid metabolism‐related pathways from tissue RNA‐seq (Adj normal tissues: *n* = 9; PCa: *n* = 18) of Zhongnan Hospital cohort (Adj normal vs PCa samples). (B) Correlation analysis of 8 key genes with lipid metabolism‐related pathways in PCa, ordered from left to right according to the size of the absolute value of the correlation coefficient. Positive values (red) indicate positive correlations, negative values (blue) indicate negative correlations with the indicated lipid metabolism–related pathways. The magnitude of the correlation coefficient reflects the strength of the association. (C,D) Representative pictures of plate cloning experiments and Transwell assays after transfected with the respective plasmids for 48 h in PC‐3 cells (C) and the corresponding statistical graphs (D) (*n* = 3). Scale bar = 5 mm. (E) *GSTM4* mRNA expression levels in PCa (*n* = 36) and normal tissues (*n* = 23) from PCa patients at Zhongnan Hospital. (F) The *GSTM4* mRNA expression level in PCa (*n* = 18) and normal tissues (*n* = 9) from tissue RNA‐seq of the Zhongnan Hospital cohort. (G) Representative immunohistochemical images and the GSTM4 staining score of PCa (*n* = 44) and paired adjacent tissues (*n* = 44) in a tissue microarray (HProA150PG02 cohort). Scale bar = 200 µm. (H) Protein expression levels of GSTM4 in seven randomly selected pairs of PCa tissues and corresponding adjacent tissues. (I) Representative Immunohistochemical images of GSTM4 in four randomly selected pairs of PCa tissues and corresponding adjacent tissues (*n* = 4). Scale bar = 100 µm. (J) Statistics on the correlation between GSTM4 RNA levels and triglyceride content in tissues of PCa patients in the Zhongnan Hospital cohort (*n* = 39). (K) The *GSTM4* mRNA expression level in TCGA‐PRAD cohort. (L) The mRNA level of *GSTM4* in different gleason score in TCGA‐PRAD cohort. M) Kaplan‐Meier curves for PCa patients with high and low GSTM4 expression in the TCGA‐PRAD cohort (log rank test, *p* = 0.0069). A group with High GSTM4 mRNA levels and a group with low GSTM4 mRNA levels were categorized based on the median GSTM4 expression. *p* values were determined by paired Student's t‐tests (G), two‐tailed unpaired Student's t test (E, F, and K), and one‐way ANOVA with Dunnett's multiple comparisons (D and L). The data are shown as the means ± SDs.

### 2.2 GSTM4 Inhibits the Proliferation and Migration of PCa Cells

We assessed GSTM4 protein expression across various PCa cell lines and a normal prostate cell line (RWPE‐1). GSTM4 expression was lower in PCa cell lines (LNCaP, DU145, PC‐3, 22RV1) compared to the normal prostate cell line RWPE‐1 (Figure 2A). Among the cancer cell lines, DU145 exhibited the highest GSTM4 expression, while PC‐3 had the lowest. Consequently, DU145 and PC‐3 were selected for subsequent functional studies. To strengthen the reliability of our findings, we performed both GSTM4 overexpression and knockdown experiments in DU145 and PC‐3 cells. This bidirectional approach enabled a comprehensive assessment of GSTM4 function while reducing the potential influence of cell line–specific effects. Three stable GSTM4 knockdown cell lines were generated, and knockdown efficiency was confirmed via Western blot analysis (Figure 2B). The shGSTM4‐1 construct, demonstrating the most efficient silencing, was used in downstream experiments. The CCK‐8 and Plate cloning assays demonstrated that GSTM4 knockdown enhanced the proliferation of PCa cell lines (Figure 2C,D). Additionally, Transwell and wound healing assays revealed that GSTM4 knockdown also enhanced cell migration in PCa cell lines (Figure 2E and Figure S3A). Subsequently, we established PC‐3 and DU145 cell lines with stable overexpression of GSTM4 and validated the expression by Western blot (Figure 2F). In contrast, GSTM4 overexpression inhibited both proliferation and migratory capabilities (Figure 2G–I and Figure S3B). Given that DU145 and PC‐3 are androgen receptor (AR)‐negative prostate cancer cell lines, we further validated the biological function of GSTM4 in AR‐positive prostate cancer cells. To this end, key phenotypic assays, including colony formation and Transwell migration assays, were performed in 22Rv1 and LNCaP cells. Consistent with our observations in AR‐negative cell lines, GSTM4 overexpression significantly suppressed cell growth and migration, whereas GSTM4 depletion markedly promoted these malignant phenotypes (Figure S3C–J). These results further support the tumor‐suppressive role of GSTM4 in prostate cancer regardless of AR status.

**FIGURE 2:**
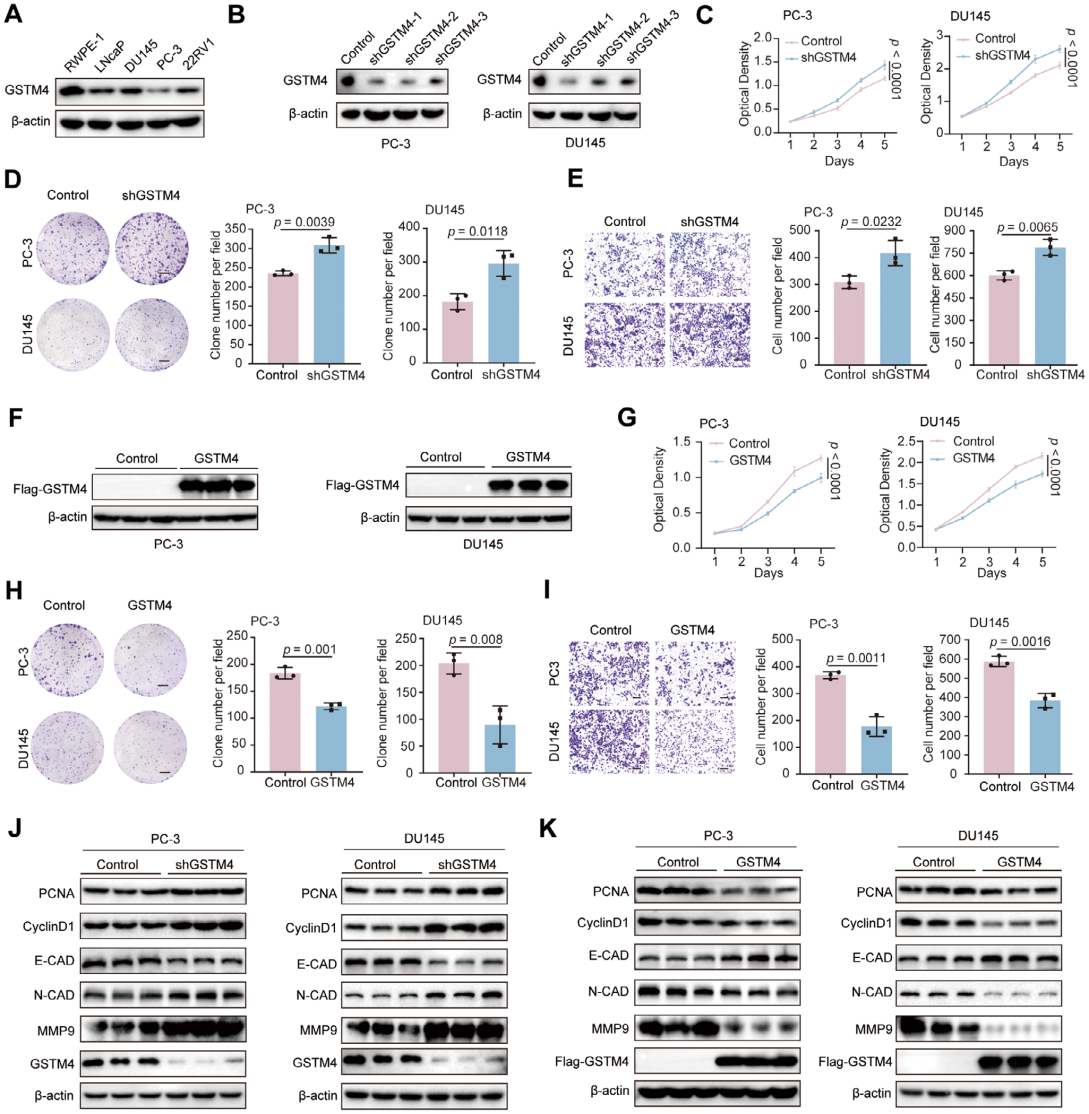
GSTM4 inhibits the proliferation and migration of PCa cells. (A) GSTM4 protein expression in normal prostate cells and different PCa cell lines was detected by Western blot analysis (*n* = 3). (B) Validation of GSTM4 stable knockdown in PC‐3 and DU145 cell lines (*n* = 3). (C) The viability of PC‐3 and DU145 cells with GSTM4 stable knockdown was determined by CCK‐8 assays (*n* = 3). (D) Representative pictures of plate cloning experiments after stable knockdown of GSTM4 in the PCa cells and the corresponding statistical graphs (*n* = 3). Scale bar = 5 mm. (E) Representative images of Transwell assays after stable knockdown of GSTM4 in the PCa cells and the corresponding statistical graphs (*n* = 3). Scale bar = 50 µm. (F) Validation of GSTM4 stable overexpression in PC‐3 and DU145 cell lines (*n* = 3). (G) The viability of PC‐3 and DU145 cells with GSTM4 stable overexpression was determined by CCK‐8 assays (*n* = 3). (H) Representative pictures of plate cloning experiments after stable overexpression of GSTM4 in the PCa cells and the corresponding statistical graphs (*n* = 3). Scale bar = 5 mm. (I) Representative images of Transwell assays after stable overexpression of GSTM4 in the PCa cells and the corresponding statistical graphs (*n* = 3). Scale bar = 50 µm. (J,K) Western blot assay was performed to detect changes in proliferation‐associated proteins and EMT‐associated proteins in the GSTM4 stable knockdown (J) or overexpression (K) cell lines PC‐3 and DU145, and β‐actin was used as an internal control protein (n = 3). *p* values were determined by two‐tailed unpaired Student's t test (C,D,E,G–I). The data are shown as the means ± SDs.

Western blot analyses supported these findings. GSTM4 knockdown led to an upregulation of proliferation markers such as PCNA and Cyclin D1, as well as epithelial–mesenchymal transition (EMT)‐related proteins including N‐cadherin and MMP9. Conversely, E‐cadherin expression decreased (Figure 2J). Overexpression of GSTM4 resulted in opposite effects (Figure 2K and Figure S4A–D). These results highlight that GSTM4 plays a crucial role in inhibiting the proliferation and migration of PCa cells.

### 2.3 GSTM4 Inhibits the Proliferation, Migration and Lipid Metabolism of PCa Cells Independently of Its Enzymatic Activity

To determine whether the inhibitory effect of GSTM4 on the proliferation, migration of PCa cells is attributed to the enzymatic activity of GSTM4. We mutated the tyrosine at position 7 of GSTM4 to alanine to remove the glutathione transferase function of GSTM4 [26]. The absence of glutathione transferase activity was determined by measuring its GSH content (Figure 3A). Unfortunately, the following plate cloning assays and Transwell assay showed that loss of enzymatic activity did not restore its inhibitory effect on PCa cell proliferation and migration (Figure 3B,C).

**FIGURE 3:**
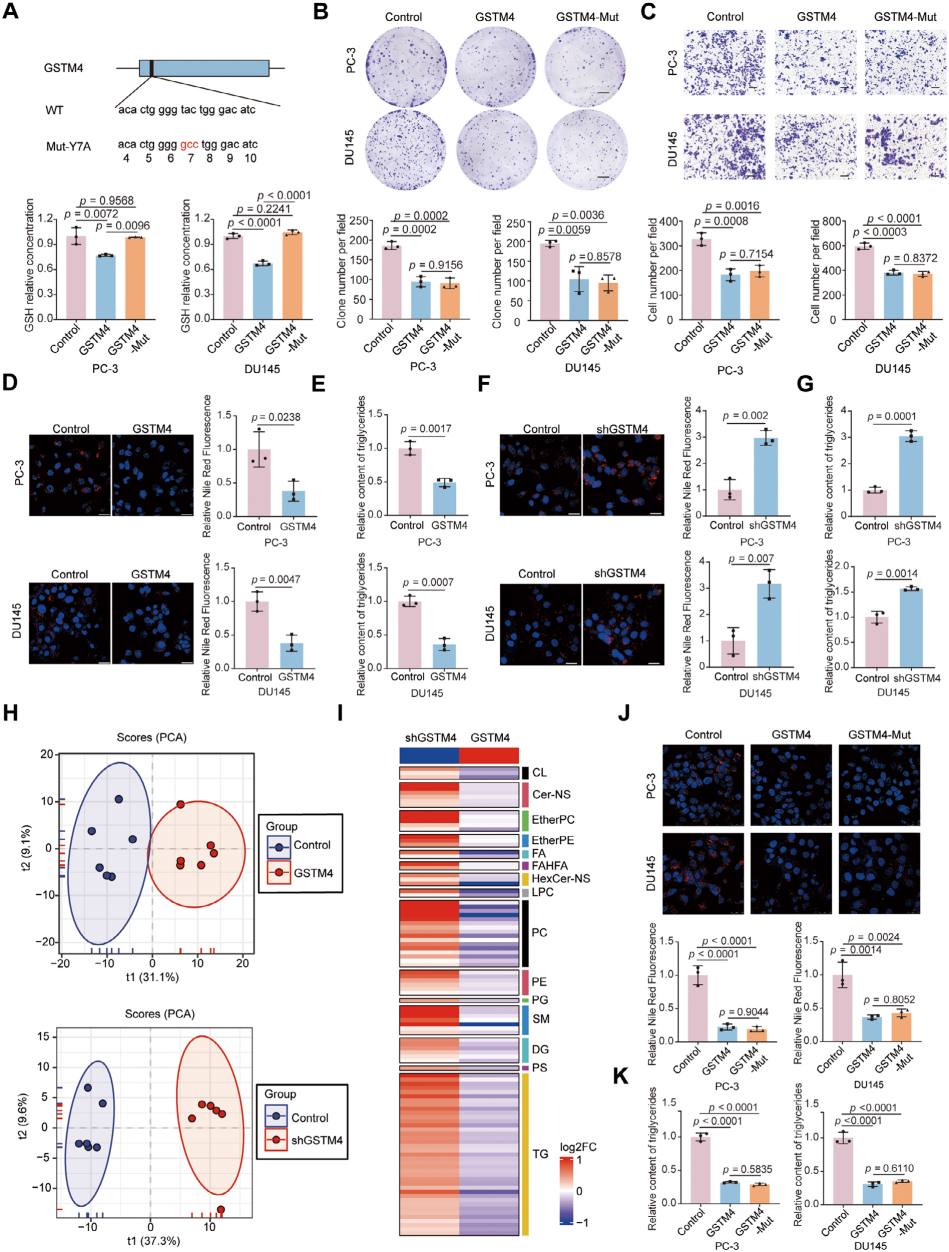
GSTM4 inhibits the proliferation, migration, and lipid synthesis of PCa cells independently of its enzymatic activity. (A) Schemas of GSTM4 enzyme active mutant plasmid construction (top) and statistical graphs of relative intracellular GSH content (bottom) (*n* = 3). (B) Representative images (top) and statistical results (bottom) of plate cloning experiments of control, GSTM4 stable overexpression cell line, and GSTM4‐mut stable overexpression cell line (*n* = 3). Scale bar = 5 mm. (C) Representative images (top) and statistical results (bottom) of Transwell assays in control and GSTM4 stable overexpression cell lines and GSTM4‐mut stable overexpression cell lines (*n* = 3). Scale bar = 50 µm. (D) Representative images of Nile red staining experiments (left) with GSTM4 stably overexpressing cell lines and the corresponding statistical graphs (right) in PC‐3 (top) and DU145 cell (bottom) lines (*n* = 3). Scale bar = 25 µm. (E) Relative triglyceride content from stable GSTM4 overexpression in PC‐3 (top) and DU145 cell (bottom) lines (*n* = 3). (F) Representative images of Nile red staining experiments (left) with GSTM4 stably knockdown cell lines and the corresponding statistical graphs (right) in PC‐3 (top) and DU145 cell (bottom) lines (*n* = 3). Scale bar = 25 µm. (G) Relative triglyceride content from stable GSTM4 knockdown in PC‐3 (top) and DU145 cell (bottom) lines (*n* = 3). (H) PCA (Principal Component Analysis) of untargeted lipidomics in the GSTM4 stable overexpression (top) or knockdown (bottom) cell lines PC‐3 (*n* = 6). (I) Untargeted lipidomics in the GSTM4 stable overexpression and knockdown cell lines PC‐3 (*n* = 6). (J) Representative images (top) and statistical results (bottom) of Nile red staining experiments in the control group, GSTM4 stable overexpression cell line, and GSTM4‐mut stable overexpression cell line (*n* = 3). Scale bar = 25 µm. (K) Statistical results of relative intracellular triglyceride content in PC‐3 (left) and DU145 (right) cells (*n* = 3). *p* values were determined by two‐tailed unpaired Student's t test (D–G) and one‐way ANOVA with Dunnett's multiple comparisons (A,B,C,J, and K). The data are shown as the means ± SDs.

Interestingly, stable overexpression of GSTM4 significantly reduced lipid droplet accumulation (Figure 3D) and triglyceride content (Figure 3E) in both PC‐3 and DU145 cell lines. Conversely, GSTM4 knockdown increased lipid droplet levels (Figure 3F) and triglyceride content (Figure 3G). Similar results were observed in the AR‐positive prostate cancer cell lines 22Rv1 and LNCaP, where GSTM4 overexpression significantly decreased triglyceride content, whereas GSTM4 knockdown resulted in elevated triglyceride levels (Figure S4E,F). Given the established link between GSTM4 and lipid metabolism, we performed untargeted lipidomic analysis on PC‐3 cells with stable GSTM4 knockdown or overexpression. The PCA plot showed clear separation among the compared groups, indicating good reproducibility of the lipidomics data (Figure 3H). Lipidomic profiling demonstrated that GSTM4 overexpression markedly reduced levels of various lipid species, including triglycerides (TG) and fatty acids (FA), while GSTM4 knockdown resulted in a significant increase in these lipids (Figure 3I), quantitative data for the identified lipid species are summarized in Table S1. Furthermore, Nile Red staining and triglyceride content assays corroborated that the inhibitory effect of GSTM4 on lipid metabolism (Figure 3J,K) was independent of its glutathione transferase activity.

### 2.4 GSTM4 Interacts With ACLY and Reduces ACLY Protein Level in PCa Cells

To explore the molecular mechanisms by which GSTM4 influences lipid metabolism, we performed mass spectrometry analysis on PC‐3 cell lines. The proteins bound to GSTM4, identified through mass spectrometry, were cross‐referenced with lipid synthesis‐related proteins to isolate the candidate target proteins, FASN and ACLY (Figure 4A). Co‐immunoprecipitation (Co‐IP) experiments demonstrated that GSTM4 does not interact with FASN (Figure 4B). Subsequent endogenous and exogenous Co‐IP results confirmed that GSTM4 interacts with ACLY (Figure 4C,D). GST‐pulldown assays substantiated the direct interaction between GSTM4 and ACLY (Figure 4E). Immunofluorescence co‐localization experiments demonstrated that GSTM4 co‐localized with ACLY within the cytoplasm (Figure 4F). The RNA levels of ACLY remained unchanged following either knockdown or overexpression of GSTM4 (Figure S5A–D). However, Immunohistochemical analysis of ACLY in paired cancerous and adjacent cancerous prostate tissue samples revealed a significantly elevated expression of ACLY in PCa (Figure 4G and Figure S5E). Immunohistochemical staining of tissue microarrays also revealed markedly increased ACLY protein levels in paired PCa samples (Figure S5F,G). Furthermore, stratification analysis based on Gleason score demonstrated that ACLY expression progressively increased with tumor grade, whereas GSTM4 expression gradually decreased (Figure S5H–J). In agreement with these observations, GSTM4 expression was negatively correlated with ACLY expression in clinical specimens (Figure S5K). Western blot experiments confirmed that overexpression of GSTM4 significantly reduced the protein levels of ACLY (Figure 4H and Figure s5 L–N). Conversely, knockdown of GSTM4 produced the opposite effect (Figure 4I and Figure S5O–Q). Collectively, these findings suggest that GSTM4 interacts with ACLY and suppresses its protein expression, thereby contributing to the regulation of lipid metabolism in PCa cells.

**FIGURE 4:**
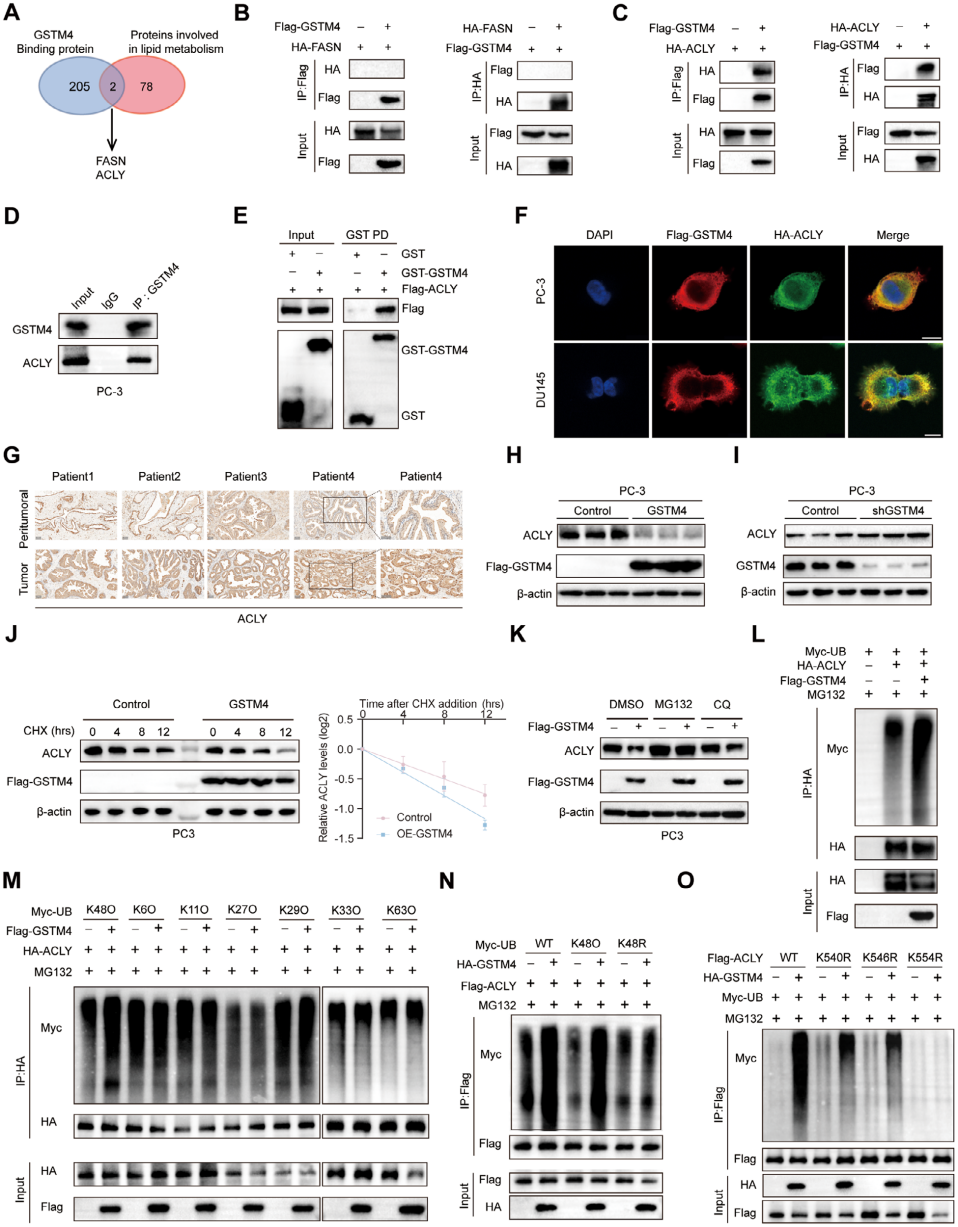
GSTM4 interacts with ACLY and enhances its ubiquitination at K554, thereby promoting ACLY degradation and reducing its protein level. (A) Two proteins were identified as overlapping between GSTM4 binding proteins (207) and lipid metabolism related proteins (80) in PC‐3 cells. (B) 293T cells were transfected with the respective plasmids for 48 h, and IP experiments were performed using anti‐Flag or anti‐HA antibodies. (C) 293T cells were transfected with the respective plasmids for 48 h, and IP experiments were performed using anti‐Flag or anti‐HA antibodies. (D) IP assay with GSTM4 antibody in 293T cell lysates to verify the interaction between GSTM4 and ACLY. (E) A GST pull‐down assay was conducted to verify the direct interaction between GSTM4 and the ACLY protein in vitro. (F) IF assays were used to detect the expression and localization of GSTM4‐Flag (red) and ACLY‐HA (green) in PC‐3 (top) and DU145 cells (bottom), and nuclei were stained with DAPI (blue). Scale bar = 10 µm. (G) Representative immunohistochemical staining of ACLY in tumor tissue sections (*n* = 4) from Zhongnan Hospital. Scale bar = 50 µm. (H,I) Western blot assay was performed to detect changes in the protein levels of ACLY in the GSTM4 stable overexpression (H) or knockdown (I) cell lines PC‐3, and β‐actin was used as an internal control protein (*n* = 3). (J) Western blot assays were conducted by transfecting the respective plasmids into PC‐3 cells for 48 h and incubating them with CHX (50 µg/mL) for the indicated times (*n* = 3). Representative images of the Western blot assays (left) and the corresponding protein densitometry analysis (right). (K) PC‐3 cells were transfected with the respective plasmids and treated with DMSO, MG132 (10 µM), or CQ (25 µM) for 8 h for Western blot assays. (L) The respective plasmids were transfected into 293T cells for 48 h, and MG132 (10 µM) was added for 8 h. Anti‐HA antibody was added to perform the ubiquitination assays. M) The respective plasmids were transfected into 293T cells for 48 h, MG132 (10 µM) was added for 8 h. Ubiquitination assays were performed to detect the specific type of ubiquitin chain linked to ACLY affected by GSTM4. (N) The indicated plasmids were transfected into 293T cells for 48 h, and MG132 (10 µM) was added for 8 h. Anti‐Flag antibody was added to perform the ubiquitination assays. (O) The respective plasmids were transfected into 293T cells for 48 h. MG132 (10 µM) was added for 8 h. Anti‐Flag antibody was added to perform the ubiquitination assays.

### 2.5 GSTM4 Enhances Ubiquitination of ACLY at K554 to Promote its Degradation

Previous data demonstrated a direct interaction between GSTM4 and ACLY, with GSTM4 overexpression leading to reduced ACLY protein levels. Notably, this reduction occurred without affecting *ACLY* mRNA expression (Figure S5A–D), suggesting that GSTM4 regulates ACLY through post‐translational mechanisms. To explore this hypothesis, we investigated the impact of GSTM4 on ACLY protein stability in PCa cell lines. Our findings indicate that overexpression of GSTM4 accelerates the degradation of ACLY in PCa cell lines (Figure 4J and Figure S5R–T). The application of MG132 completely reversed this effect (Figure 4K and Figure S5U), corroborating previous reports that ACLY protein undergoes degradation via the ubiquitin–proteasome pathway. Ubiquitination assays further confirmed that GSTM4 markedly enhances the ubiquitination of ACLY (Figure 4L). These results suggest that GSTM4 facilitates the ubiquitination and subsequent degradation of ACLY. To identify the specific type of ubiquitin chain involved, we constructed seven ubiquitin mutants (K6O, K11O, K27O, K29O, K33O, K48O, and K63O), each retaining only one lysine residue while the remaining lysines were mutated to arginine. Subsequent ubiquitination experiments showed that GSTM4 induced K48‐linked ubiquitination of ACLY (Figure 4M). Furthermore, mutation of the K48 residue to arginine (K48R) abolished the effect of GSTM4 on ACLY ubiquitination (Figure 4N), confirming the specificity of the K48 linkage in this process. As expected, GSTM4 enhances K48‐linked ubiquitination of ACLY, indicating an elevated susceptibility of ACLY to proteasomal degradation. To pinpoint the exact ubiquitination site on ACLY affected by GSTM4, we mutated ACLY at residues K540, K546, and K554 to arginine (K540R, K546R, K554R) based on previous references [27]. Ubiquitination assays revealed that GSTM4 failed to promote ubiquitination of the ACLY‐K554R mutant (Figure 4O), whereas the other mutants remained susceptible. In conclusion, our findings indicate that GSTM4 enhances K48‐linked ubiquitination of ACLY specifically at residue K554, thereby promoting the ubiquitin‐proteasome degradation of ACLY.

### 2.6 GSTM4 Affects the Progression of PCa by Regulating ACLY In Vitro

To verify whether GSTM4 inhibits the progression of PCa through the down‐regulation of ACLY, we performed plate cloning assay and a Transwell assay after overexpression of GSTM4 and ACLY, respectively or simultaneously, in PCa cells (PC‐3 and DU145). Overexpression of ACLY in the cell line stably overexpressing GSTM4 significantly restored its proliferation and migration ability (Figure 5A–D). Furthermore, we utilized the FDA‐approved ACLY inhibitor, ETC‐1002. This allowed us to assess if inhibiting ACLY could counteract the PCa progression induced by GSTM4 knockdown. Plate cloning experiments (Figure 5E,F) and Transwell experiments (Figure 5G,H) demonstrated that ECT‐1002 significantly restored the proliferation and migration of GSTM4 stable knockdown cell lines. To investigate the impact on lipid metabolism, Nile Red staining (Figure S6A) and triglyceride quantification assays (Figure S6B) were performed. Overexpression of ACLY significantly increased intracellular lipid droplets and triglyceride content, effectively reversing the depletion of lipid droplets and the reduction in triglyceride levels caused by GSTM4 overexpression (Figure S6A,B). In contrast, treatment with ETC‐1002 produced the opposite effect (Figure S6C,D).

**FIGURE 5:**
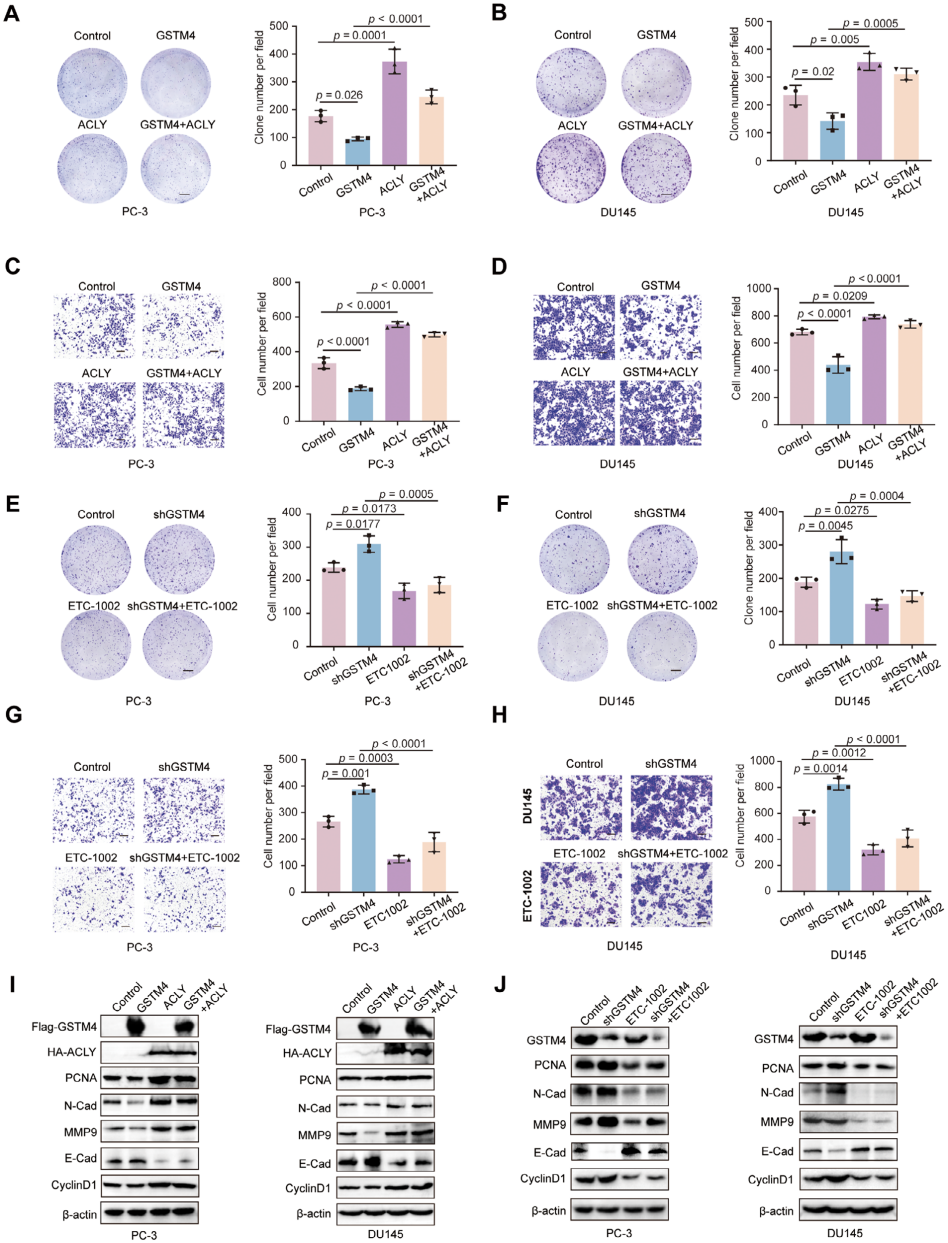
GSTM4 affects the progression of PCa by regulating ACLY in vitro. (A,B) Representative images and statistical analysis of plate cloning assays in PC‐3 (A) and DU145 (B) cells overexpression of GSTM4 and ACLY, respectively or simultaneously (*n* = 3). Scale bar = 5 mm. (C,D) Representative images and statistical analysis of Transwell assays in PC‐3 (C) and DU145 (D) cells overexpression of GSTM4 and ACLY, respectively or simultaneously (n = 3). Scale bar = 50 µm. E‐F) Representative images and statistical analysis of plate cloning assays in PC‐3 (E) and DU145 (F) cells (Control or shGSTM4) treated with or without ETC‐1002 (50 µmol/L) for 48 h (*n* = 3). Scale bar = 5 mm. (G,H) Representative images and statistical analysis of Transwell assays in PC‐3 (G) and DU145 (H) cells (Control or shGSTM4) treated with or without ETC‐1002 (50 µmol/L) for 48 h (*n* = 3). Scale bar = 50 µm. (I) Western blot assay was performed to detect changes in proliferation‐associated proteins and EMT‐associated proteins in PC‐3 (left) and DU145 (right) cells overexpression of GSTM4 and ACLY, respectively or simultaneously, and β‐actin was used as an internal control protein. (J) Western blot assay was performed to detect changes in proliferation‐associated proteins and EMT‐associated proteins in PC‐3 (left) and DU145 (right) cells (Control or shGSTM4) treated with or without ETC‐1002 (50 µmol/L) for 48 hr, and β‐actin was used as an internal control protein. *p* values were determined by one‐way ANOVA with Dunnett's multiple comparisons. The data are shown as the means ± SDs.

Western blot analyses were conducted to validate these phenotypic observations. The overexpression of GSTM4 led to decreased levels of proliferation markers (PCNA, Cyclin D1) and EMT markers (N‐cadherin, MMP9), along with elevated E‐cadherin expression, supporting earlier findings (Figure 2K). Furthermore, the overexpression of ACLY reversed these alterations in GSTM4 overexpression cells (Figure 5I). In contrast, supplementation with ETC‐1002 in GSTM4 stable knockdown cells yielded the opposite effect (Figure 5J).

### 2.7 GSTM4, E3 Ubiquitin Ligase TRIM27, and ACLY Exhibit Mutual Interaction

Our previous findings demonstrated that GSTM4 directly interacts with ACLY, facilitating its ubiquitination and promoting proteasomal degradation. However, GSTM4 lacks a canonical E3 ubiquitin ligase domain, and there is no existing evidence supporting its role in direct regulation of protein stability. This led us to hypothesize that GSTM4 may promote ACLY ubiquitination by recruiting or interacting with other E3 ubiquitin ligases or deubiquitinating enzymes. To investigate this, we cross‐referenced proteins identified through GSTM4 mass spectrometry analysis with known E3 ligases and deubiquitinating enzymes, identifying seven candidate proteins (Figure 6A,B). We constructed expression plasmids for each candidate E3 ligase—UPF1, WDR33, PRPF19, TRIM27, CORO2A, PWP2, and PDLIM2—and assessed their effect on ACLY expression. Of these, only CORO2A, TRIM27, and PRPF19 were found to reduce ACLY protein levels (Figure 6C). Co‐IP experiments further confirmed that only TRIM27, among these three, interacts with ACLY (Figure 6D). Subsequent Co‐IP experiments confirmed the interaction between GSTM4 and TRIM27, which was consistent with the results of mass spectrometry analyses (Figure 6E,F). GST pull‐down experiments verified the direct interaction between GSTM4, TRIM27, and ACLY (Figure 6G,H). Immunofluorescence analysis indicated their co‐localization in the cytoplasm (Figure S7A,B). To confirm whether GSTM4 enhances K48‐linked ubiquitination of ACLY at residue K554 via TRIM27, we performed ubiquitination assays. The results showed that TRIM27 enhances K48‐linked ubiquitination of ACLY (Figure 6I). Further assays with ACLY point mutations revealed that TRIM27 specifically increases ubiquitination at the K554 site of ACLY (Figure 6J), consistent with the effect of GSTM4 on ACLY ubiquitination.

**FIGURE 6:**
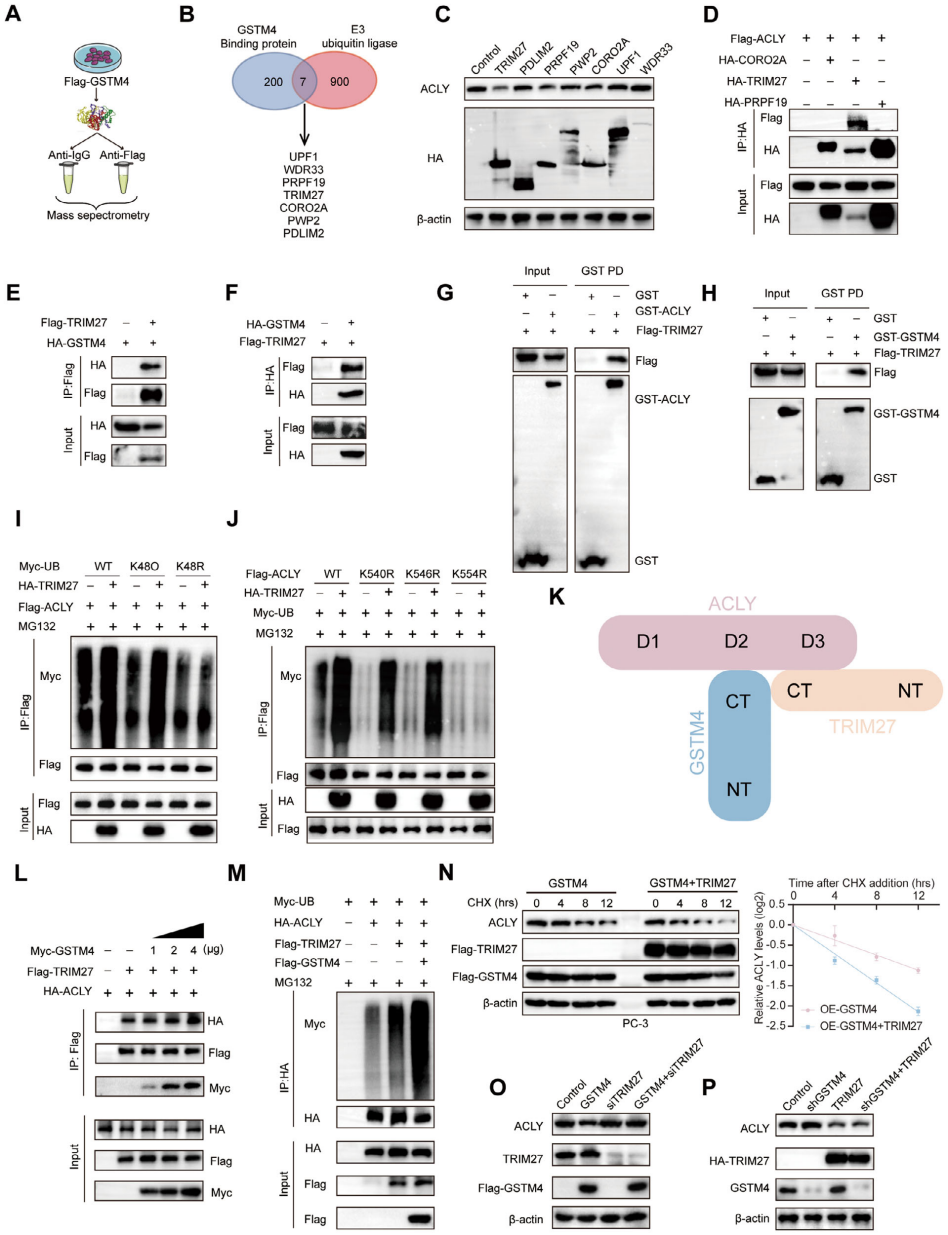
GSTM4, E3 ubiquitin ligase TRIM27, and ACLY exhibit mutual interaction, GSTM4 enhancing the ubiquitination of ACLY by TRIM27. (A) Flow chart for mass spectrometry analysis of PC‐3 cells stably overexpressing GSTM4 and control cells. (B) Seven proteins were identified as overlapping between GSTM4 binding proteins (207) and E3 ubiquitin ligase‐related proteins (907) in PC‐3 cells. (C) The respective plasmids were transfected into PC‐3 cells for 48 h, and Western blot assay was performed to detect the expression of ACLY. (D) The respective plasmids were transfected into PC‐3 cells for 48 h, and IP experiments were performed using anti‐HA antibodies. (E,F) 293T cells were transfected with the respective plasmids for 48 h, and IP experiments were performed using anti‐Flag (E) or anti‐HA (F) antibodies. (G) A GST pull‐down assay was conducted to verify the direct interaction between TRIM27 and the ACLY protein in vitro. (H) A GST pull‐down assay was conducted to verify the direct interaction between TRIM27 and the GSTM4 protein in vitro. (I) The indicated plasmids were transfected into 293T cells for 48 h, and MG132 (10 µM) was added for 8 h. Ubiquitination assays were performed to detect ubiquitination of ACLY protein. (J) The respective plasmids were transfected into 293T cells for 48 h, and MG132 (10 µM) was added for 8 h. An anti‐Flag antibody was added to perform the ubiquitination assays. (K) Pattern diagram of the complex formed by GSTM4, TRIM27, ACLY. (L) 293T cells were transfected with the respective plasmids for 48 h, and quantitative IP experiments were performed using anti‐Flag antibodies. (M) The respective plasmids were transfected into 293T cells for 48 h. MG132 (10 µM) was added for 8 h. An anti‐HA antibody was added to perform the ubiquitination assays. (N) Western blot assays were conducted by transfecting the respective plasmids into stable overexpression of GSTM4 in the PC‐3 cells for 48 h and incubating them with CHX (50 µg/mL) for the indicated times (*n* = 3). Representative images of the Western blot assays (left) and the corresponding protein densitometry analysis (right). (O) GSTM4‐Flag plasmids and siTRIM27 RNA were transfected into PC‐3 cells for 48 h, and Western blot assay was performed to detect the expression of ACLY. (P) TRIM27‐HA plasmids were transfected into PC‐3 cells (Control or shGSTM4 group) for 48 h, and Western blot assay was performed to detect the expression of ACLY.

To identify the specific binding regions, we constructed truncated plasmids for ACLY (D1: 1 – 440, D2: 441 – 778, D3: 779 – 1101), TRIM27 (DN: 1 – 127, DC: 128 – 513), and GSTM4 (DN: 1 – 88, DC: 89 – 218) (Figure S7C). Subsequent IP experiments revealed that the D2 region of ACLY interacts with the C‐terminus of GSTM4 (Figure S7D). The C‐terminus of GSTM4 interacts with the C‐terminus of TRIM27 (Figure S7E), while the C‐terminus of TRIM27 binds to the D3 region of ACLY (Figure S7F). These findings suggest that GSTM4, TRIM27, and ACLY form an interactive complex (Figure 6K). As an E3 ubiquitin ligase, TRIM27 also enhances substrate ubiquitination. Based on these results, we have identified TRIM27 as a key candidate molecule.

### 2.8 GSTM4 Enhances TRIM27‐Mediated Ubiquitination of ACLY

In PC‐3 and DU145 cells, overexpression of TRIM27 inhibited ACLY protein expression without affecting GSTM4 protein levels (Figure S7G,H). However, GSTM4 overexpression did not alter TRIM27 protein levels (Figure S7I,J).

These findings suggest that GSTM4's influence on ACLY is independent of changes in TRIM27 protein levels, and the interaction between TRIM27 and GSTM4 does not impact GSTM4 protein levels. To further investigate this interaction, co‐transfection of GSTM4, TRIM27, and ACLY in 293T cells was followed by Co‐IP assays. Results showed that increasing doses of transfected GSTM4 plasmids enhanced the binding affinity of TRIM27 to ACLY (Figure 6L). Subsequent ubiquitination assays confirmed that GSTM4 amplifies TRIM27‐mediated ubiquitination of ACLY (Figure 6M). This key result suggests that GSTM4 strengthens the interaction between TRIM27 and ACLY, thereby boosting TRIM27's ubiquitination activity on ACLY.

To further validate the conclusion that GSTM4 regulates ACLY stability via TRIM27, we overexpressed TRIM27 in PC‐3 cells stably overexpressing GSTM4. Overexpression of TRIM27 in GSTM4 stable overexpression PC‐3 cells further decreased ACLY protein stability (Figure 6N). Knockdown of TRIM27 using siRNA while stably overexpressing GSTM4 restored the inhibition of ACLY by overexpressing GSTM4 (Figure 6O). In contrast, stable knockdown of GSTM4 with concomitant overexpression of TRIM27 significantly reversed the promotion of ACLY expression by knockdown of GSTM4 (Figure 6P). To further determine whether the enzymatic activities of GSTM4 and TRIM27 are involved in the regulation of ACLY, we performed enzymatic activity–deficient mutant assays. The results showed that TRIM27 enhanced K48‐linked ubiquitination of ACLY in an E3 ubiquitin ligase activity‐dependent manner, whereas the catalytic mutant of TRIM27 (TRIM27‐C16A/C31A) failed to promote ACLY ubiquitination (Figure S8A). In contrast, loss of GST transferase activity did not affect the regulatory function of GSTM4 toward ACLY. Notably, the enzymatically inactive GSTM4‐Y7A mutant retained its ability to facilitate the interaction between TRIM27 and ACLY (Figure S8B), consistent with the phenotypic observations. Collectively, these findings indicate that GSTM4 regulates the TRIM27–ACLY axis independently of its enzymatic activity, whereas the E3 ligase activity of TRIM27 is required for ACLY ubiquitination. In summary, GSTM4 enhances K48‐linked ubiquitination of ACLY at residue K554 through TRIM27, thereby reducing ACLY protein stability.

### 2.9 GSTM4 Inhibits PCa Proliferation and Lipid Synthesis In Vivo by Affecting ACLY

To further validate the role of GSTM4 in PCa proliferation in vivo, subcutaneous xenografts and prostate orthotopic xenografts models were established in nude mice. In subcutaneous xenografts models, GSTM4 knockdown significantly accelerated tumor growth, as indicated by increased tumor volume and weight (Figure 7A–C). In contrast, GSTM4 overexpression markedly suppressed tumor development (Figure 7D–F). Subcutaneous tumor tissues were collected for Western blot analysis to assess the expression of proteins related to proliferation and EMT. Consistent with the in vitro results, the shGSTM4 group showed increased levels of ACLY, PCNA, Cyclin D1, N‐cadherin, and MMP9, while E‐cadherin levels decreased compared to the control group (Figure S9A,B). In contrast, overexpression of GSTM4 produced the opposite effects (Figure S9C,D). Hematoxylin and eosin (H&E) staining of the subcutaneous xenografts revealed greater histological heterogeneity in the shGSTM4 group compared to the control group (Figure 7G). Immunohistochemical analysis showed that GSTM4 knockdown led to an increase in Ki67‐positive proliferating cells and heightened ACLY staining intensity (Figure 7G and Figure S9E). Furthermore, Oil Red O staining of the shGSTM4 tumors indicated substantial lipid droplet accumulation within the tumor cells (Figure 7G). In contrast, overexpression of GSTM4 produced the opposite effects (Figure 7H and Figure S9F). As with the subcutaneous xenograft model, in vivo inhibition of PCa proliferation and lipid synthesis by GSTM4 was also demonstrated in orthotopic xenograft models of PCa (Figure S9G–L).

**FIGURE 7:**
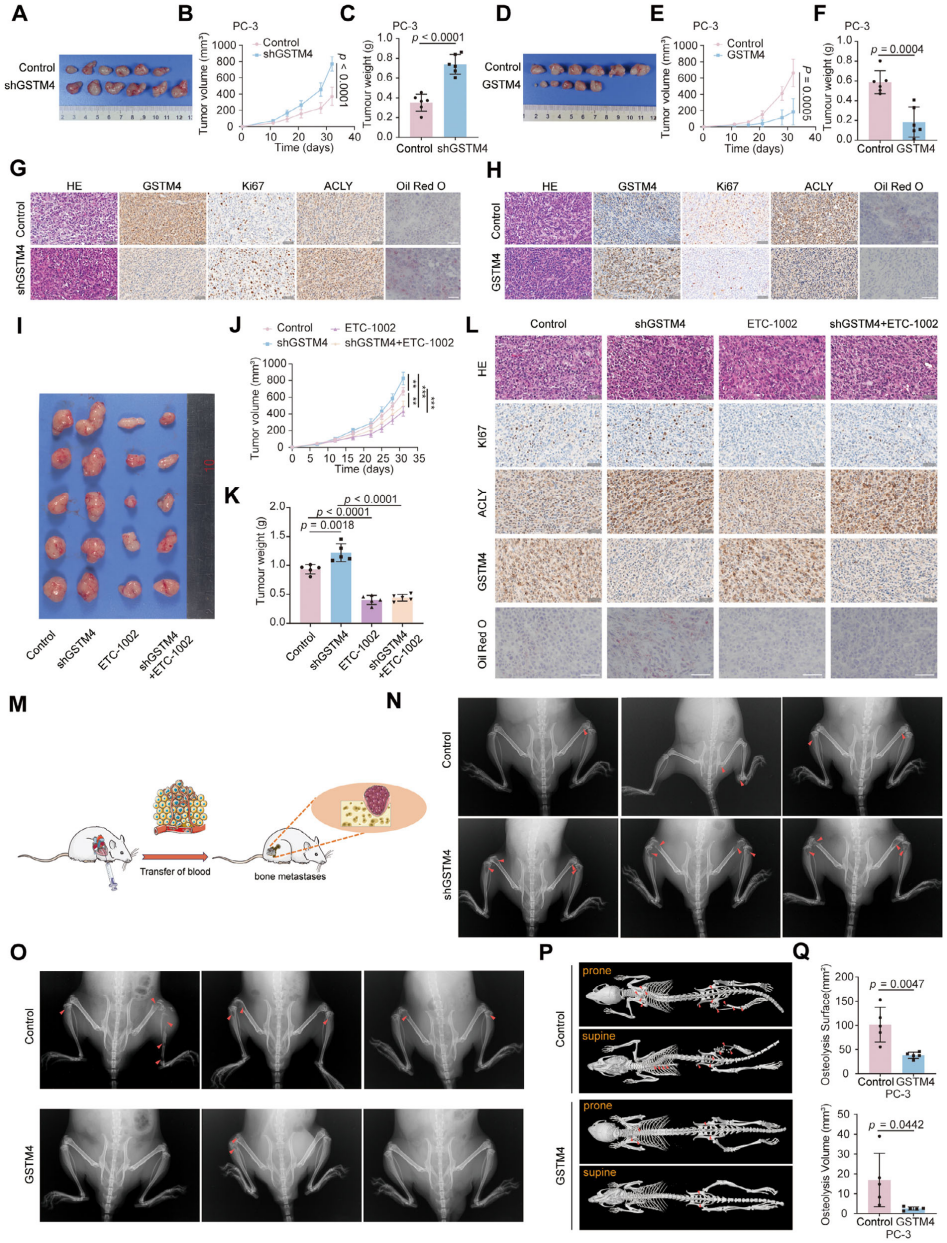
GSTM4 inhibits PCa proliferation, lipid synthesis, and bone metastasis in vivo by affecting ACLY. (A–C) Gross view (A) and volume (B)and weight (C) statistics of tumors in the PC‐3 cell line subcutaneous xenograft model (control group vs. shGSTM4 group, n = 6 per group). (D–F) Gross view (D) and volume (E) and weight (F) statistics of tumors in the PC‐3 cell line subcutaneous xenograft model (control group vs. GSTM4 stable overexpression group, *n* = 6 per group). (G) Representative images of H&E staining analysis, Immunohistochemical (GSTM4, Ki67, ACLY), and Oil red O staining analysis in xenograft tumors of PC‐3 cells (control group vs. shGSTM4 group, Scale bar = 50 µm). (H) Representative images of H&E staining analysis, Immunohistochemical (GSTM4, Ki67, ACLY), and Oil red O staining analysis in xenograft tumors of PC‐3 cells (control group vs. GSTM4 stable overexpression group, Scale bar = 50 µm). (I–K) Gross view (I) and volume (J) and weight (K) statistics of tumors in the PC‐3 cell line subcutaneous xenograft model (control group; shGSTM4 group; ETC‐1002 group; shGSTM4 group + ETC‐1002 group). *n* = 5 per group. (L) Representative images of H&E staining analysis, Immunohistochemical (Ki67, ACLY, GSTM4), and Oil red O staining analysis in xenograft tumors of PC‐3 cells (control group; shGSTM4 group; ETC‐1002 group; shGSTM4 group + ETC‐1002 group), Scale bar = 50 µm. (M) Schematic representation of a PCa bone metastasis model constructed by left ventricular injection of PC‐3 cells with stable GFP expression in NOD/SCID mice. (N) Representative x‐ray images of the control and shGSTM4 groups 45 days after injection (*n* = 5 per group). (O) Representative X‐ray images of the control and GSTM4 stable overexpression group 60 days after injection (*n* = 5 per group). (P,Q) Representative images of CT three‐dimensional reconstruction of bone (P) and statistics of osteolysis area (Q, top) and volume (Q, bottom) in the control group and stable overexpression GSTM4 group 60 days after injection (*n* = 5 per group). *p* values were determined by two‐tailed unpaired Student's t test (B,C,E,F, and Q) and one‐way ANOVA with Dunnett's multiple comparisons (J,K). The data are shown as the means ± SDs.

To further confirm that GSTM4 inhibits PCa proliferation and lipid synthesis by down‐regulating ACLY in vivo, we constructed four groups of subcutaneous xenograft mice (Control, shGSTM4, ETC‐1002, shGSTM4+ETC‐1002). The results showed that ETC‐1002 could effectively inhibit the growth of PCa in nude mice. The tumor volume and weight of the shGSTM4+ ETC‐1002 group were significantly lower than those of the control group or the shGSTM4 group (Figure 7I–K). H&E staining and Ki67 staining of subcutaneous xenograft tissues revealed a significant decrease in heterogeneity and proliferative capacity in the shGSTM4+ETC‐1002group compared to the shGSTM4 group (Figure 7L). Oil red staining showed that ETC‐1002 could reduce the content of lipid droplets in PCa tissues (Figure 7L). Taken together, these results confirm that GSTM4 inhibits PCa proliferation and lipid synthesis by down‐regulating ACLY expression in vivo.

### 2.10 GSTM4 Inhibits Bone Metastasis of PCa In Vivo

A PCa bone metastasis model was successfully established by injecting GFP‐tagged PC‐3 cells into the left ventricle of NOD/SCID mice to investigate the effect of GSTM4 on PCa bone metastasis in vivo (Figure 7M). Compared with the control group, there was significant osteolysis of the distal femur and proximal tibia in the shGSTM4 group of mice, while a decrease in bone density was observed by X‐ray imaging (Figure 7N). H&E staining and GFP fluorescence microimaging confirmed bone metastasis of PCa cells at the site of osteolysis. Immunohistochemical results also confirmed that ACLY expression in PCa cells at the site of bone metastasis increased with GSTM4 knockdown (Figure S10A).

In contrast, X‐ray imaging revealed a significant reduction in osteolytic areas in the GSTM4 overexpression group compared to the control group (Figure 7O). Three‐dimensional skeletal reconstruction via CT further confirmed a marked decrease in both the area and volume of osteolysis in the overexpression group (Figure 7P,Q). H&E staining and GFP fluorescence microscopy confirmed the successful establishment of the bone metastasis model. Immunohistochemical analysis of bone tissues showed a significant reduction in ACLY expression at the PCa metastatic sites in the GSTM4 overexpression group (Figure S10B).

## 3 Discussion

Our study uncovers a novel role for GSTM4 in regulating lipid metabolism and the progression of prostate cancer (PCa). We demonstrate that GSTM4 suppresses de novo lipid synthesis by promoting the ubiquitination and degradation of ATP citrate lyase (ACLY), a key enzyme in fatty acid biosynthesis. This function is independent of GSTM4's classical enzymatic activity in detoxification, highlighting a non‐canonical role in tumor biology. Lipid metabolic reprogramming is a hallmark of cancer cells, facilitating rapid proliferation and survival under metabolic stress [28]. In PCa, enhanced lipogenesis and lipid accumulation are associated with tumor aggressiveness and poor clinical outcomes [29, 30]. Our data align with these observations, showing that inhibition of lipid synthesis via GSTM4 upregulation suppresses PCa cell growth and migration, adding to the growing body of literature emphasizing the critical role of lipid metabolism in PCa progression [10, 31].

The tumor microenvironment (TME) plays a pivotal role in cancer development and progression, influencing tumor cell behavior through complex interactions with stromal cells, immune cells, and extracellular matrix components [32]. Metabolic alterations within the TME can modulate cancer cell metabolism, promoting adaptation to nutrient‐deprived conditions and supporting malignant phenotypes [33]. In PCa, the periprostatic adipose tissue is a significant component of the TME, providing fatty acids and other metabolites that fuel tumor growth [34]. Our findings that GSTM4 modulates lipid metabolism highlight the interplay between tumor cells and their microenvironment, suggesting that GSTM4 may influence how PCa cells respond to metabolic cues within the TME Figure 8.

**FIGURE 8:**
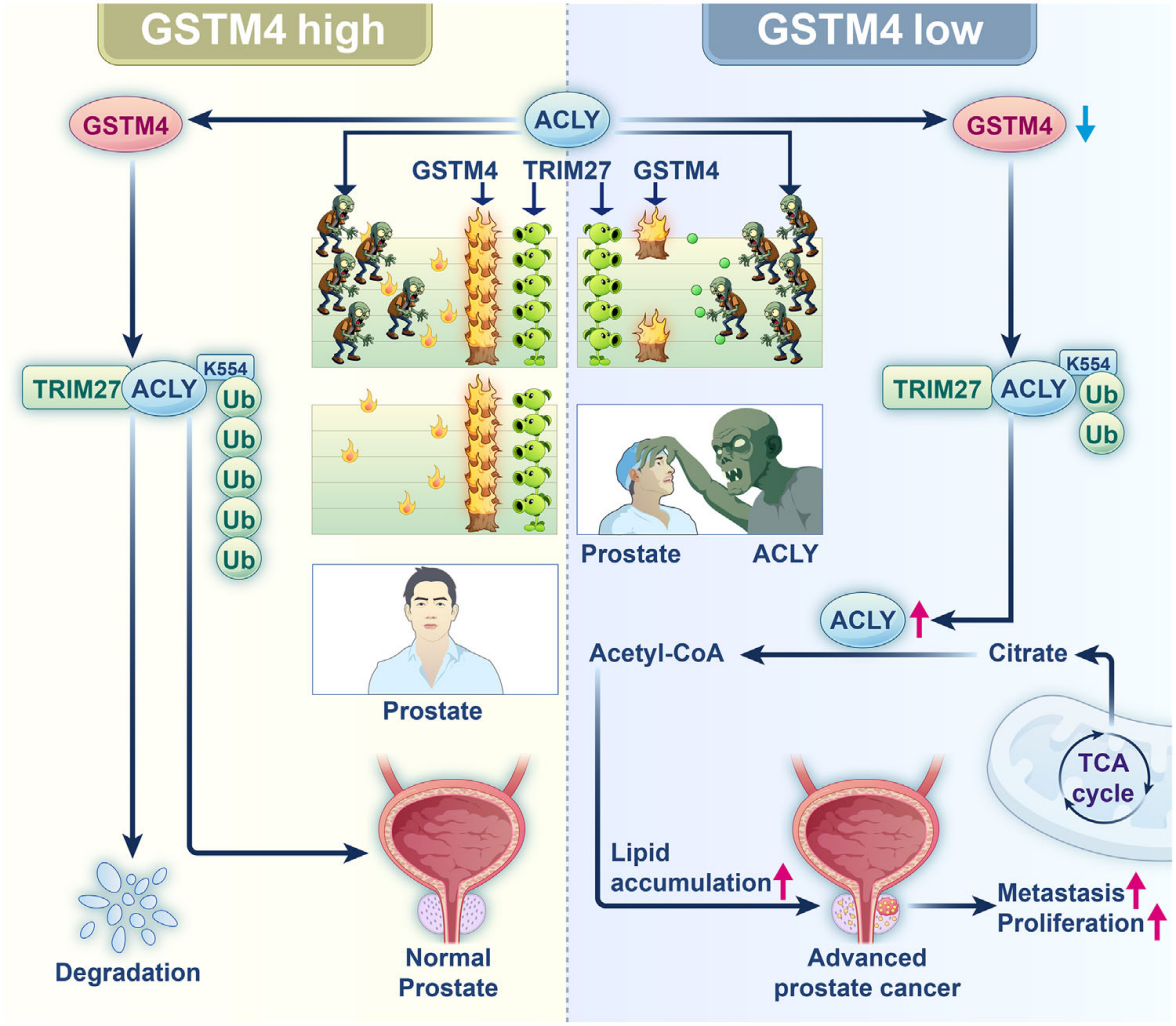
Mechanistic diagram of this study. GSTM4 enhances the interaction between ACLY and the E3 ubiquitin ligase TRIM27, thereby increasing K48‐linked ubiquitination at the K554 site of ACLY and promoting its degradation. Consequently, GSTM4 regulates lipid synthesis, as well as the proliferation and metastasis of PCa cells, by modulating ACLY stability.

The identification of GSTM4 as a regulator of lipid metabolism in PCa is particularly noteworthy. While glutathione S‐transferases (GSTs) are traditionally recognized for their roles in detoxification and drug resistance [22], emerging evidence suggests they have broader functions in cellular signaling and metabolic regulation [35]. GSTM4, belonging to the mu class of GSTs, has been less studied in the context of cancer. Our findings that GSTM4 negatively correlates with triglyceride content and impacts lipid metabolic pathways expand the understanding of its function beyond detoxification.

Mechanistically, we found that GSTM4 forms a complex with tripartite motif‐containing 27 (TRIM27) and ACLY, enhancing TRIM27‐mediated ubiquitination of ACLY at lysine 554. ACLY is a pivotal enzyme linking glucose metabolism to lipid synthesis by converting citrate to acetyl‐CoA [36]. Its overexpression has been implicated in various cancers, including PCa, promoting lipogenesis and tumor growth [37, 38]. By facilitating ACLY degradation, GSTM4 effectively reduces acetyl‐CoA availability for fatty acid synthesis, thereby inhibiting lipid accumulation and tumor progression. This mechanism underscores the significance of ubiquitin‐mediated proteolysis in controlling metabolic enzymes and highlights the interplay between protein ubiquitination and metabolic regulation in cancer cells. The involvement of TRIM27, which possesses E3 ubiquitin ligase activity and is implicated in transcriptional regulation and innate immunity [39, 40], adds another layer to the regulatory network. Furthermore, our study reveals that the tumor‐suppressive effects of GSTM4 are independent of its glutathione transferase activity, consistent with reports that certain GST family members and other enzymes exert non‐enzymatic functions influencing cell signaling pathways [25, 41]. Mechanistically, TRIM27 requires its E3 ligase activity to ubiquitinate ACLY. In contrast, GSTM4 acts primarily as a scaffold. It enhances the physical interaction between TRIM27 and ACLY without relying on its own enzymatic function.

The metabolic adaptability of cancer cells is crucial for survival within the nutrient‐deprived and hypoxic conditions of the TME [25]. By altering lipid metabolism, GSTM4 may impact not only tumor cell proliferation but also their ability to withstand metabolic stress. Additionally, lipid metabolites can influence signaling pathways involved in cell growth, apoptosis, and metastasis [42, 43, 44, 45]. Therefore, GSTM4's regulation of lipid metabolism could have broader implications for PCa progression and resistance to therapy. Therapeutically, our findings suggest that enhancing GSTM4 expression or mimicking its function could be a viable strategy to suppress lipid synthesis and inhibit PCa progression. Targeting metabolic enzymes has gained traction in cancer therapy, with several inhibitors of lipid metabolism currently under investigation [46, 47]. Disrupting protein‐protein interactions within the GSTM4–TRIM27–ACLY complex may offer another therapeutic avenue. Moreover, modulating the metabolic interactions between tumor cells and their microenvironment could enhance the efficacy of existing treatments [48, 49]. Combining metabolic inhibitors with immunotherapies or chemotherapies may overcome resistance mechanisms mediated by the TME [50, 51], and understanding how GSTM4 influences these interactions could inform combination strategies to improve clinical outcomes.

However, translating these findings into clinical applications requires careful consideration. The systemic inhibition of ACLY could have adverse effects due to its essential role in normal cellular metabolism [52]. Therefore, strategies that selectively target cancer cells, perhaps by exploiting differences in GSTM4 expression or activity, would be advantageous. Additionally, understanding the regulation of GSTM4 itself could provide insights into how its expression or activity is altered in PCa. Our study has limitations that warrant further investigation. The precise molecular determinants governing the interaction between GSTM4, TRIM27, and ACLY remain to be elucidated, and structural studies could provide deeper insights into the binding interfaces, facilitating the design of molecules that modulate these interactions. Furthermore, while our in vitro and in vivo models demonstrate the functional significance of GSTM4, clinical studies are necessary to validate its role and assess its potential as a biomarker or therapeutic target in PCa patients.

In conclusion, we present evidence that GSTM4 acts as a tumor suppressor in PCa by promoting the degradation of ACLY, thereby inhibiting lipid synthesis and tumor progression. This study highlights a novel non‐enzymatic role of GSTM4 and underscores the importance of metabolic regulation and tumor microenvironment interactions in cancer. Targeting the GSTM4‐TRIM27‐ACLY axis offers a promising strategy for the development of new therapies against PCa.

## 4 Experimental Model and Subject Details

### 4.1 Tissue Samples

Human prostate cancer (PCa) tissue samples used in this study were provided by the Department of Urology, Zhongnan Hospital of Wuhan University (Tables S2–S6). The study was approved by the institutional ethics review board (Approval No. 2021125). In this study using tissue microarray (HProA150PG02) was purchased from Outdo Biotech (Suzhou, China).

### 4.2 Cell Lines

Human PCa cell lines (PC‐3, DU145, 22RV1, and LNCaP), along with non‐cancerous human prostate epithelial cells (REPE‐1) and embryonic kidney cells (HEK293T), were cultured in standard growth medium containing 10% fetal bovine serum and 1% penicillin‐streptomycin (Table S7). Transfections of siRNAs and plasmids were performed using Lipofectamine 3000 (L3000015, Invitrogen) in accordance with the manufacturer's guidelines. All cell lines were verified to be mycoplasma‐free via DNA testing.

### 4.3 Method Details

#### 4.3.1 Triglyceride Quantification

Triglyceride content in cells and tissues was assessed following the manufacturer's guidelines using a triglyceride quantitation kit. In cell experiments, cells were collected and suspended in 200 µL PBS, then sonicated on ice to produce cell homogenates. Protein concentration was measured within the same homogenate sample, and the triglyceride content was normalized relative to the protein content. For tissue assays, 50 mg of clinical samples from 39 prostate cancer (PCa) patients (Table S3) were weighed and combined with 450 µL absolute ethanol to create tissue homogenates. The details of chemicals and critical commercial assays used in this subject are listed in Table S8.

#### 4.3.2 Nile Red Staining and Oil Red O Staining

For Nile Red Staining, cells were seeded onto coverslips, fixed with 4% paraformaldehyde for 30 mins at room temperature, and stained with Nile Red (2 µg/mL) and DAPI (0.5 µg/mL) for 15 mins. Imaging was conducted with a confocal laser microscope, and lipid accumulation was quantified using ImageJ software. For Oil Red O staining, cryosections of OCT‐embedded subcutaneous tumor tissues were stained following standard histological protocols.

#### 4.3.3 siRNAs and shRNA

The siRNAs used for this study were purchased from GenePharma (Suzhou, China). The siRNA sequences are listed in Table S9. The shRNAs used in this study were synthesized by Tsingke (Beijing, China), and the shRNA sequences are listed in Table S9.

### 4.4 Construction of Plasmid and Lentivirus Stable Cell Lines

In this study, GSTM4, ACLY, TRIM27, PDLIM2, PRPF19, CORO2A, PWP2 plasmids were constructed through the standard method of homologous recombination. UPF1 and WDR33 plasmids were procured from Miaoling Biology (Wuhan, China). GSTM4 knockdown and overexpression plasmids were generated using a lentivirus vector. The lentivirus plasmid, along with two packaging plasmids, pMD2.G and psPAX2, were co‐transfected into HEK29T cells for 48 h, and the supernatant was collected and filtered. Following infection, 4 µg/mL puromycin was added 36 h later for cell line selection. The primer sequences used are detailed in Table S10.

### 4.5 CCK8 Cell Proliferation Assay

The CCK8 proliferation assay was performed according to the experimental protocol of the CCK8 kit (Bimake, USA). The treated cells were counted and plated in 96‐well plates at 2500 cells per well. After cell adhesion, 10% CCK8 solution was given on the first, second, third, fourth, and fifth day, and the cells were incubated in the cell incubator for 2 h in the dark. Cell OD values were determined at a wavelength of 450 nm using a Multiskan GO.

### 4.6 Colony Formation Assays

Treated cells were seeded into 6‐well plates at a density of 1,000 cells per well. After cell attachment (approximately 3 days), the culture medium was replaced. Colonies were allowed to grow for 1–2 weeks before fixation with 4% paraformaldehyde for 1 h. Cells were then stained using 800 µL of 0.1% crystal violet per well for another 1 h. Finally, the wells were rinsed with distilled water, air‐dried, imaged, and colony numbers were quantified using Image‐Pro Plus software.

### 4.7 Wound Healing Assays

For the wound healing assays, treated cells were seeded in six‐well plates and subjected to overnight serum starvation following excessive cell growth. Linear wounds were drawn and photographed at the same location at 0 h (F1) and 24 h (F2). The wound distance was then measured, and the wound healing rate was calculated using the formula: (F1‐F2) / F1 * 100%.

### 4.8 Transwell Assays

For the Transwell assays, the treated cells (8 × 10^4^ PC‐3 or 4 × 10^4^ DU145 cells) were counted and resuspended using 100 µL of medium (serum‐free) and added to the top of the chamber. While 600 µL of complete medium was added to the bottom of the chamber. After 24 h of incubation, the chambers were fixed with 4% paraformaldehyde for half an hour and stained with 0.1% crystal violet for an additional hour. After the chamber was washed, five random fields were photographed, and the number of cells was counted by Image‐Pro Plus.

### 4.9 Western Blotting

Total proteins were extracted from cultured cells or tissue samples using RIPA buffer supplemented with protease and phosphatase inhibitors. Protein concentration was determined using the BCA assay (B34304, Beyotime). Equal amounts of protein were resolved by SDS‐PAGE (10%) and transferred onto PVDF membranes. Membranes were blocked with skim milk and incubated sequentially with primary and secondary antibodies. Bands were visualized using an ECL kit and imaged with a chemiluminescence detection system. Antibodies used are listed in Table S11.

### 4.10 RNA Extraction and Quantitative Reverse Transcription PCR (qRT‐PCR)

Total RNA extraction, reverse transcription, and quantitative PCR were performed as previously described [53]. The sequences of primers used are detailed in Table S12.

### 4.11 Coimmunoprecipitation (co‐IP) Assays and Ubiquitination Assay

Co‐IP assays were conducted using the BeaverBeadsTM Protein A/G Immunoprecipitation Kit (22202‐100, Beaver). Cells were lysed on ice with IP binding buffer containing PMSF and phosphatase inhibitors. After centrifugation, the supernatant was divided into input group, IgG control group, and IP group, where the input group was heated to boiling after adding 5×loading buffer. In IgG and IP groups, the corresponding antibodies were added and incubated at 4°C overnight. After centrifugation again, 20 µL of the beads were added to the supernatant and incubated for 2 h. The magnetic beads four times with IP binding buffer, and bound proteins were eluted with loading buffer and analyzed by Western blot.

For the ubiquitination assay, cells were treated with 10 µM MG132 (Sigma) for 6 h before collection and lysed with RIPA lysis buffer containing PMSF and phosphatase inhibitor on ice. The supernatant was divided into input and IP groups, and subsequent IP experiments and WB experiments were carried out as described above.

### 4.12 Mass Spectrometry Analysis

PC‐3 cells stably expressing GSTM4 and the control cells with an empty vector (1 × 10^7 cells each) were collected for immunoprecipitation (IP) assays and subsequent protein separation by SDS‐PAGE. The gel was silver stained according to the instructions of the silver staining kit (P0017S, Beyotime). The successful expression of GSTM4 protein was verified by WB, SDS‐PAGE gel segments were excised and sent to Shanghai Luming Biological Technology Co., Ltd. (Shanghai, China) for further analysis.

### 4.13 Untargeted Lipidomic Analysis

Stable GSTM4‐overexpressing, GSTM4‐knockdown, and control PC‐3 cells were harvested for LC‐MS‐based untargeted lipidomic profiling, performed by Shanghai Luming Biotechnology Co., Ltd. Principal component analysis (PCA) was performed using the normalized lipidomics dataset to evaluate the overall distribution of samples and assess the variability among experimental groups. Prior to PCA, lipid abundance data were log2‐transformed and normalized. The first two principal components (PC1 and PC2) were used for visualization of sample clustering and group separation. Differentially expressed lipid species were defined as those with *p* < 0.05 and fold change ≥ 1.2 or ≤ 0.83.

### 4.14 GST Pull‐Down

Expression plasmids encoding Flag or GST fusion proteins were transformed into Escherichia coli BL21 cells and purified using Ni^2^^+^‐NTA agarose. GST pull‐down assays were conducted according to manufacturer protocols. Briefly, 50 µg of GST‐tagged GSTM4, ACLY, or GST alone was incubated with glutathione Sepharose 4B beads, then mixed with 20 µg of purified Flag‐TRIM27, Flag‐ACLY, respectively. Protein interactions were analyzed by WB using anti‐FLAG and GST antibodies.

### 4.15 GSH Assay

Cellular GSH levels were determined using a total glutathione assay kit (Beyotime, S0052) following the supplier's protocol. Absorbance was read at 412 nm, and reduced glutathione content was calculated as: total GSH − 2× oxidized GSH.

### 4.16 Immunohistochemistry

Following tissue fixation in formalin and subsequent paraffin embedding, sections were cut, and dewaxing was performed. Dehydration was carried out in an ethanol gradient. Antigen retrieval was performed using a sodium citrate solution. Subsequent incubation with primary and secondary antibodies ensued. Visualization was achieved using 3,3’‐diaminobenzidine (DAB) solution. Details of the antibodies used are provided in Table S12.

### 4.17 Immunofluorescence

Cells were seeded in six‐well plates with cell slides at the bottom, maintaining a cell density of 20% for immunofluorescence co‐localization imaging. Following fixation with 4% paraformaldehyde, cells underwent three washes with PBS and were treated with 0.4% Triton X‐100 for 10 min. After another three washes with PBS, cell slides were sealed with 2% BSA for 30 mins and washed twice with PBS. The cell slides were then incubated with primary and secondary antibodies and 0.5 µg/mL DAPI. Images were captured using a confocal laser microscope. Information regarding the antibodies used is provided in Table S12.

### 4.18 Animal Experiments

For subcutaneous tumor bearing experiments, BALB/c‐nude mice were acclimated to the barrier environment for one week. Stable GSTM4 knockdown or overexpression PC‐3 cells (1×10^6^ cells/100 µL, *n* = 6) were injected subcutaneously into BALB/c‐nude mice. The mice were observed twice a week and euthanized after 32 days. Tumors were weighed and photographed and then divided into triplicate. One portion was snap‐frozen in liquid nitrogen, one was fixed in formalin, and the remaining portion was embedded using OCT.

$$\mathrm{Tumor}\;\mathrm{volume}=\mathrm{widt}h^{2}\times \frac{\mathrm{length}}{2}$$

For the subcutaneous tumor recovery experiment, BALB/c‐nude mice were randomly divided into four groups: control+solvent group, shGSTM4+solvent group, control+ECT‐1002 group, shGSTM4+ECT‐1002 group, and shGSTM4+ECT‐1002 group. Administration was started when the subcutaneous tumor volume reached 100 mm^3^. ECT‐1002 (30 mg/kg, dissolved in DMSO and diluted in PBS) and solvent were administered via oral gavage every other day for 3 weeks. The body weight of the mice was measured and the state of the mice was observed. The volume of subcutaneous tumor was measured by vernier caliper.

For the orthotopic prostate tumor bearing experiment, BALB/c‐nude mice were anesthetized with isoflurane, and stable GSTM4 knockdown or overexpression PC‐3 cells (1 × 10^6^ cells/30 µL, *n* = 5) were slowly injected into the anterior lobe of the prostate, and the medial and lateral skin of the abdomen was sutured. 30 days later, Mouse prostate tumor tissues were harvested after euthanizing and subsequently, the tissues were weighed and photographed.

PCa bone metastasis model: before injection, GFP‐labeled PC‐3 cells were suspended in 0.1 mL PBS at a density of 5 × 10^5^ to avoid cell cluster formation. Four to six‐week‐old male NOD/SCID mice were anesthetized and cardiac injected in the supine position (*n* = 5). A 30‐gauge needle was inserted 2 mm to the left of the sternum in the second intercostal space to aim at the center of the rib, and continuous slow injections of the cell suspension were made when pulsatile blood appeared at the hub of the needle. The status of the mice was observed after the completion of the injection, and fluorescence was detected to assess the formation of bone metastases. X‐ray and CT were taken to detect the area and volume of osteolytic bone. The knockdown group was euthanized after 45 days, and the overexpression group was euthanized after 60 days after injection. According to the X‐ray results, the bone with bone metastasis sites was selected for sampling.

All mice experiments were reviewed and approved by the Laboratory Animal Welfare and Ethics Committee of Zhongnan Hospital, conducted in accordance with its regulations (Approval No. ZN2022274).

### 4.19 CT Scanning

Mice were anesthetized using 3% isoflurane and 1.5% for maintenance. The sample was scanned by Micro‐CT 1276 (Germany, Belgium) with image pixels of 20um, scanning voltage of 85 kV, scanning current of 200 µA, and exposure time of 384 ms. After scanning, NRecon was used for sample reconstruction, CTAn for data analysis, and CTvox for data modeling.

### 4.20 Statistics

All statistical analyses were performed using GraphPad Prism (version 9). Data are shown as means ± SDs. Unpaired two‐tailed Student's *t*‐tests were used for comparisons between two unpaired groups, while one‐way ANOVA with Dunnett's multiple comparison test was applied for multiple comparisons. Survival curves were evaluated using the log‐rank test. Significance levels are represented as follows: ^*^: *p* < 0.05; ^**^: *p* < 0.01; ^***^: *p* < 0.001; N.S.: non‐significant (*p* > 0.05).

## Author Contributions

K.X., X.W., H.W., Y.L., and F.L. designed the experiments. K.X., F.L., W.W., K.Q., and G.W., performed the experiments. K.X., W.W., and H.W. performed the data analyses. K.X., H.W., F.L., and G.W. wrote the manuscript. S.T., S.C., H.X., and S.Z. provided technical support. H.W. and X.H.W. organized and supervised the study.

## Conflicts of Interest

The authors declare no conflicts of interest.

## Supporting information




**Supporting File**: advs77394‐sup‐0001‐SuppMat.docx.

## Data Availability

The data that support the findings of this study are available from the corresponding author upon reasonable request.
