# Supplementary material for: GSTM4 Suppresses Prostate Cancer Progression by Targeting TRIM27‐mediated ACLY Ubiquitination and Lipid Metabolism
Source: Adv Sci (Weinh). 2026 Aug 30:e77394. Online ahead of print. doi: 10.1002/advs.77394 (PMC13527044; doi:10.1002/advs.77394)
Supplement: Supplementary file 1 [file ADVS-9999-e77394-s001.docx]

Supplementary Information

GSTM4 suppresses prostate cancer progression by targeting TRIM27-mediated ACLY ubiquitination and lipid metabolism

Figures S1-S10: Pages 2-16

Tables S1-S11: Pages 17-36

Figure S1. Plasmid Validation

(A-B) Western blot assays were conducted by transfecting the respective plasmids into the PC3 (A) cells and DU145 (B) cells for 48 hrs.

Figure S2. GSTM4 was downregulated in PCa and negatively correlated with patient prognosis

(A-B) Representative pictures of plate cloning experiments and Transwell assays after transfected with the respective plasmids for 48 hrs in the DU145 cells (A) and the corresponding statistical graphs (B) (n = 3). Scale bar = 50 μm.

(C) Quantification results for immunohistochemical of GSTM4 in four randomly selected pairs of PCa tissues and corresponding adjacent tissues. Average optical density (AOD) = integral optical density (IOD) / area. The quantitative densitometry analysis of proteins was assessed with ImageJ software.

(D) The *GSTM4* mRNA expression level in GSE88808, GSE32571, GSE62872, GSE70768.

(E) The mRNA level of *GSTM4* in different Pathological N-staging and T-staging in TCGA-PRAD cohort.

*p* values were determined by one-way ANOVA with Dunnett’s multiple comparisons (B), paired Student's t-tests (C) and two-tailed unpaired Student's t test (D). The data are shown as the means ± SDs.

Figure S3. GSTM4 inhibits the proliferation and migration of PCa cells

(A) Representative images of wound healing experiments after stable knockdown of GSTM4 in the PCa cells and the corresponding statistical graphs (n = 3). Scale bar = 50 μm. (B) Representative images of wound healing experiments after stable overexpression of GSTM4 in the PCa cells and the corresponding statistical graphs (n = 3). Scale bar = 50 μm. (C-D) Validation of GSTM4 stable knockdown in 22RV1 (C) and LNCaP (D) cell lines (n = 3). (E) Representative pictures of plate cloning experiments after stable knockdown of GSTM4 in the PCa cells and the corresponding statistical graphs (n = 3). Scale bar = 5 mm. (F) Representative images of Transwell assays after stable knockdown of GSTM4 in the PCa cells and the corresponding statistical graphs (n = 3). Scale bar = 200 μm. (G-H) Validation of GSTM4 stable overexpression in 22RV1 (G) and LNCaP (H) cell lines (n = 3). (I) Representative pictures of plate cloning experiments after stable overexpression of GSTM4 in the PCa cells and the corresponding statistical graphs (n = 3). Scale bar = 5 mm. (J) Representative images of Transwell assays after stable overexpression of GSTM4 in the PCa cells and the corresponding statistical graphs (n = 3). Scale bar = 200 μm. *p* values were determined by two-tailed unpaired Student's t test (A, B, E, F, I, and J). The data are shown as the means ± SDs.

Figure S4. GSTM4 inhibits the lipid metabolism of AR-positive PCa cells

(A-B) Quantification results for proliferation-associated proteins and EMT-associated proteins in the GSTM4 stable knockdown (A) or overexpression (B) cell lines PC-3, and β-actin was used as an internal control protein (n = 3). The quantitative densitometry analysis of proteins was assessed with ImageJ software(also in revised Figure S7A). (C-D) Quantification results for proliferation-associated proteins and EMT-associated proteins in the GSTM4 stable knockdown (C) or overexpression (D) cell lines DU145, and β-actin was used as an internal control protein (n = 3). The quantitative densitometry analysis of proteins was assessed with ImageJ software. (E) Relative triglyceride content from stable GSTM4 overexpression in 22RV1 and LNCaP cell lines (n = 3). (F) Relative triglyceride content from stable GSTM4 knockdown in 22RV1 and LNCaP cell lines (n = 3). *p* values were determined by two-tailed unpaired Student's t test (A, B, C, D, E, and F). The data are shown as the means ± SDs.

Figure S5. GSTM4 reduces ACLY protein level in PCa cells

(A-B) The mRNA levels of GSTM4 and ACLY in the PC3 (A) and DU145 (B) cells after stable overexpression of GSTM4 were analyzed by qRT-PCR (n = 3). (C-D) The mRNA levels of GSTM4 and ACLY in the PC3 (C) and DU145 (D) cells after stable knockdown of GSTM4 were analyzed by qRT-PCR (n = 3). (E) Quantification results for Immunohistochemical of ACLY in four randomly selected pairs of PCa tissues and corresponding adjacent tissues. The quantitative densitometry analysis of proteins was assessed with ImageJ software. (F-G) Representative immunohistochemical images (F) and the ACLY staining score (G) of the PCa (n =60) and paired adjacent tissues (n = 60) in tissue microarray (HProA150PG03 cohort). Scale bar = 200 μm. (H-J) Statistical plots (H-I) and representative immunohistochemical images (J) of the expression levels of ACLY (H, HProA150PG03 cohort) and GSTM4 (I, HProA150PG02 cohort) in different Gleason score. Scale bar = 200 μm. (K) 89 PCa samples were quantified and the correlation between GSTM4 and ACLY protein levels was analyzed based on Pearson test. (L-N) Western blot assay was performed to detect changes in the protein levels of ACLY in the GSTM4 stable overexpression cell lines DU145 (L), 22RV1 (M) and LNCaP (N). β-actin was used as an internal control protein (n = 3). (O-Q) Western blot assay was performed to detect changes in the protein levels of ACLY in the GSTM4 stable knockdown cell lines DU145 (O), 22RV1 (P) and LNCaP (Q). β-actin was used as an internal control protein (n = 3). (R-T) Western blot assays were conducted by transfecting the respective plasmids into DU145 (R), 22RV1 (S) and LNCaP (T) cells for 48 hrs and incubating them with CHX (50 μg/mL) for the indicated times (n = 3). Representative images of the Western blot assays (left) and the corresponding protein densitometry analysis (right). (U) DU145 cells were transfected with the respective plasmids and treated with DMSO, MG132 (10 μM), or CQ (25 μM) for 8 hrs for Western blot assays.

*p* values were determined by two-tailed unpaired Student's t test (A, B, C and D), paired Student's t-tests(E, G) and one-way ANOVA with Dunnett’s multiple comparisons (H, I). The data are shown as the means ± SDs. Average optical density (AOD) = integral optical density (IOD) / area. Protein levels were quantitatively detected with ImageJ software.

Figure S6. GSTM4 inhibits the lipid synthesis by regulating ACLY *in vitro*

(A) Representative images of Nile red staining experiments in PC-3 (left) and DU145 (right) cells overexpression of GSTM4 and ACLY, respectively or simultaneously (n = 3). Scale bar = 25 μm. (B) Statistical analysis of relative triglyceride content in PC-3 (left) and DU145 (right) cells overexpression of GSTM4 and ACLY, respectively or simultaneously (n = 3). (C) Representative images of Nile red staining experiments in PC-3 (left) and DU145 (right) cells (Control or shGSTM4) treated with or without ETC-1002 (50 μmol/L) for 48 hrs (n = 3). Scale bar = 25 μm. (D) Statistical analysis of relative triglyceride content in PC-3 (left) and DU145 (right) cells (Control or shGSTM4) treated with or without ETC-1002 (50 μmol/L) for 48 hrs (n = 3). *p* values were determined by one-way ANOVA with Dunnett’s multiple comparisons (B and D). The data are shown as the means ± SDs.

Figure S7. GSTM4, TRIM27, and ACLY exhibit mutual interaction

(A) IF assays were used to detect the expression and localization of ACLY-Flag (red) and TRIM27-HA (green) in PC-3 (top) and DU145 cells (bottom), and nuclei were stained with DAPI (blue). Scale bar = 10 μm. (B) IF assays were used to detect the expression and localization of GSTM4-Flag (red) and TRIM27-HA (green) in PC-3 (top) and DU145 cells (bottom), and nuclei were stained with DAPI (blue). Scale bar = 10 μm. (C) Design of ACLY, TRIM27 and GSTM4 truncations. (D) 293T cells were transfected with the respective plasmids for 48 hrs, and IP experiments were performed using anti-Flag or anti-HA antibodies. (E) 293T cells were transfected with the respective plasmids for 48 hrs, and IP experiments were performed using anti-Flag antibodies. (F) 293T cells were transfected with the respective plasmids for 48 hrs, and IP experiments were performed using anti-Flag or anti-HA antibodies. (G-H) TRIM27-HA plasmids were transfected into PC-3 (G) and DU145 (H) cells for 48 hrs, and Western blot assay was performed to detect the expression of ACLY and GSTM4 (n = 3). (I-J) GSTM4-Flag plasmids were transfected into PC-3 (I) and DU145 (J) cells for 48 hrs, and Western blot assay was performed to detect the expression of TRIM27 (n = 3).

Figure S8. Functional validation of GSTM4 and TRIM27 enzymatic activities in ACLY regulation

(A) PC-3 cells were transfected with the indicated plasmids for 48 hrs, followed by the addition of MG132 (10 μM) for 8 hrs. Ubiquitination assays were performed to detect polyubiquitination of the ACLY protein. (B) PC-3 cells were transfected with the indicated plasmids for 48 hrs, followed by the addition of MG132 (10 μM) for 8 hrs and quantitative IP experiments were performed using anti-Flag antibodies.

Figure S9. GSTM4 inhibits PCa proliferation *in vivo* by affecting ACLY.

(A) Western blot assay was performed to detect changes in proliferation-associated proteins and EMT-associated proteins in xenograft tumors of PC-3 Control and shGSTM4 group (n = 3). (B) Quantification results for proliferation-associated proteins and EMT-associated proteins in xenograft tumors of PC-3 GSTM4 stable knockdown (n = 3). The quantitative densitometry analysis of proteins was assessed with ImageJ software. (C) Western blot assay was performed to detect changes in proliferation-associated proteins and EMT-associated proteins in xenograft tumors of PC-3 Control and GSTM4 stable overexpression group (n = 3). (D) Quantification results for proliferation-associated proteins and EMT-associated proteins in xenograft tumors of PC-3 GSTM4 stable overexpression (n = 3). The quantitative densitometry analysis of proteins was assessed with ImageJ software. (E-F) Statistical graphs of IHC staining for GSTM4, Ki-67 and ACLY in xenograft tumors of PC-3 GSTM4 stable knockdown (E) and GSTM4 stable overexpression (F). The quantitative densitometry analysis of proteins was assessed with ImageJ software. (G) Gross view (left) and tumor weight statistics (right) of PCa orthotopic xenograft tumors (PC-3: control group vs. shGSTM4 group, n = 5 per group). (H) Gross view (left) and tumor weight statistics (right) of PCa orthotopic xenograft tumors (PC-3: control group vs. stable overexpression group, n = 5 per group). (I) Western blot assay was performed to detect changes in proliferation-associated proteins and EMT-associated proteins in PCa orthotopic xenograft tumors of PC-3 Control and shGSTM4 group (n = 3). (J) Quantification results for proliferation-associated proteins and EMT-associated proteins in PCa orthotopic xenograft tumors of PC-3 GSTM4 stable knockdown (n = 3). The quantitative densitometry analysis of proteins was assessed with ImageJ software. (K) Western blot assay was performed to detect changes in proliferation-associated proteins and EMT-associated proteins in PCa orthotopic xenograft tumors of PC-3 Control and GSTM4 stable overexpression group (n = 3). (L) Quantification results for proliferation-associated proteins and EMT-associated proteins in PCa orthotopic xenograft tumors of PC-3 GSTM4 stable overexpression (n = 3). The quantitative densitometry analysis of proteins was assessed with ImageJ software. *p* values were determined by two-tailed unpaired Student's t test (B, D, E, F, G, H, J, and L). The data are shown as the means ± SDs.

Figure S10. GSTM4 inhibits PCa bone metastasis *in vivo* by affecting ACLY.

(A) Representative images of H&E staining, immunohistochemical staining (GSTM4, ACLY), and fluorescence microscopy photography of bone metastatic sites in the control group and shGSTM4 group. Scale bar = 100 μm. (B) Representative images of H&E staining, immunohistochemical staining (GSTM4, ACLY), and fluorescence microscopy photography of bone metastatic sites in the control group and stable overexpression GSTM4 group. Scale bar = 100 μm.

Supplementary Table S1. Quantitative results of the untargeted lipidomics analysis.

| Lipidomics | shGSTM4 | OE-GSTM4 |
| --- | --- | --- |
| CL 70:3 | 0.574604157 | -0.425751915 |
| CL 72:8 | 0.111641106 | -0.286930997 |
| CL 68:2 | 0.234973921 | -0.562234248 |
| Cer_NS d40:1 | 1 | -0.112679162 |
| Cer_NS d16:1_23:0 | 1 | -0.118559096 |
| Cer_NS d19:1_24:0 | 0.550000157 | -0.148691064 |
| Cer_NS d18:1_23:0 | 0.320079784 | -0.070308318 |
| Cer_NS d18:1_25:0 | 0.179709929 | -0.128092913 |
| Cer_NS d20:1_24:0 | 0.17298169 | -0.14438036 |
| EtherPC 16:1e_22:6 | 1 | -0.180830487 |
| EtherPC 16:0e_15:0 | 1 | -0.021476961 |
| EtherPC 38:1e | 1 | -0.056820811 |
| EtherPC 38:5e | 0.249991111 | -0.028969658 |
| EtherPC 44:4e | 0.075301137 | -0.440123654 |
| EtherPE 16:1e_22:5 | 1 | -0.046897381 |
| EtherPE 18:0e_22:5 | 0.846556709 | -0.011428724 |
| EtherPE 14:1e_20:3 | 0.505585587 | -0.122775018 |
| FA 20:6 | 0.703811822 | -0.853516525 |
| FA 20:3 | 0.026137301 | -0.284403214 |
| FAHFA 20:4_20:3 | 0.940147169 | -0.053359072 |
| FAHFA 20:4_18:1 | 0.410017846 | -0.055324016 |
| HexCer_NS d36:1 | 0.593938436 | -0.157453758 |
| HexCer_NS d43:2 | 0.128334681 | -0.191761335 |
| HexCer_NS d39:1 | 0.537828075 | -1 |
| LPC 18:0/0:0 | 0.760114354 | -0.781140728 |
| LPC 0:0/26:0 | 0.046155496 | -0.407805778 |
| PC 39:5 | 1 | -0.070510371 |
| PC 16:1_18:2 | 1 | -0.752945316 |
| PC 37:4 | 1 | -0.413054331 |
| PC 15:0_16:1 | 1 | -1 |
| PC 42:7 | 0.977900277 | -0.451778602 |
| PC 17:0_20:4 | 0.600771249 | -0.198026678 |
| PC 15:0_16:0 | 0.400494866 | -0.046081784 |
| PC 38:5 | 0.517448595 | -0.179585881 |
| PC 18:1_19:1 | 0.808830135 | -0.561958136 |
| PC 18:0_20:4 | 0.210167424 | -0.013594583 |
| PC 16:0_17:1 | 0.627616033 | -0.448072571 |
| PC 32:2 | 0.755997112 | -0.63387977 |
| PC 40:5 | 0.090911197 | -0.197439101 |
| PC 14:0_17:1 | 0.419035332 | -0.527662329 |
| PC 16:0_16:2 | 0.016464658 | -0.20283092 |
| PC 29:1 | 0.12954553 | -0.346876928 |
| PE 18:1_18:3 | 0.869130781 | -0.071873581 |
| PE 17:1_18:2 | 0.781892583 | -0.255197171 |
| PE 16:1_20:4 | 0.423821265 | -0.158936214 |
| PE 38:6 | 0.277778571 | -0.098243521 |
| PE 35:1 | 0.204833996 | -0.136118511 |
| PE 16:0_16:0 | 0.043913728 | -0.05858856 |
| PG 18:1_20:1 | 0.434642952 | -0.22786528 |
| SM d33:1 | 1 | -0.228828177 |
| SM d39:1 | 1 | -0.223697528 |
| SM d41:2 | 1 | -0.093916756 |
| SM d30:1 | 0.792319779 | -0.012730974 |
| SM d32:2 | 1 | -1 |
| SM d32:1 | 0.086487093 | -0.026272709 |
| SM d41:1 | 0.200697427 | -0.174380369 |
| DG 18:0_20:3 | 0.565119896 | -0.082713792 |
| DG 18:0_22:4 | 0.589209372 | -0.23946459 |
| DG 16:0_20:4 | 0.502468292 | -0.344376127 |
| DG 18:1_20:4 | 0.261282352 | -0.158268073 |
| DG 18:0_22:3 | 0.138068961 | -0.038174847 |
| DG 16:0_16:0 | 0.058959998 | -0.277777387 |
| PS 18:1_18:1 | 0.422703461 | -0.26063583 |
| TG 52:5 | 1 | -0.352420125 |
| TG 16:1_18:1_18:2 | 0.682789615 | -0.046382157 |
| TG 16:0_18:1_20:4 | 0.904623766 | -0.284565195 |
| TG 17:0_18:0_18:0 | 0.798129511 | -0.260768881 |
| TG 55:1 | 0.611200197 | -0.145864148 |
| TG 14:0_16:1_17:1 | 0.881587206 | -0.443530813 |
| TG 16:0_18:0_20:0 | 0.553976833 | -0.143605208 |
| TG 16:0_18:0_18:0 | 0.536195289 | -0.167589887 |
| TG 51:3 | 0.752895808 | -0.389829788 |
| TG 16:0_17:1_18:1 | 0.606187198 | -0.243918622 |
| TG 53:1 | 0.535915126 | -0.189507341 |
| TG 18:0_18:0_18:1 | 0.596985093 | -0.262564047 |
| TG 16:0_17:0_18:0 | 0.719197038 | -0.391749665 |
| TG 58:3 | 0.439126295 | -0.117542413 |
| TG 53:2 | 0.570650383 | -0.252115686 |
| TG 53:3 | 0.540606173 | -0.244491364 |
| TG 48:2 | 0.569676918 | -0.27524441 |
| TG 16:0_18:0_18:1 | 0.415112777 | -0.140452698 |
| TG 16:0_18:1_18:2 | 0.3905703 | -0.117627538 |
| TG 16:0_17:0_18:1 | 0.586581716 | -0.317646474 |
| TG 55:2 | 0.468013234 | -0.221691492 |
| TG 16:0_16:0_18:1 | 0.438812658 | -0.195574617 |
| TG 18:0_18:1_20:1 | 0.467709632 | -0.243939222 |
| TG 14:0_16:0_18:1 | 0.406049901 | -0.200543202 |
| TG 55:3 | 0.436692521 | -0.23119721 |
| TG 16:0_18:1_20:3 | 0.52051521 | -0.341815354 |
| TG 16:0_16:0_17:0 | 0.530853967 | -0.369483089 |
| TG 16:0_16:0_18:0 | 0.325969724 | -0.197343316 |
| TG 54:3 | 0.797236304 | -0.68932437 |
| TG 48:3 | 0.473328904 | -0.367316371 |
| TG 16:0_16:0_16:0 | 0.308676682 | -0.237540835 |
| TG 14:0_16:0_16:0 | 0.295350495 | -0.289238304 |
| TG 14:0_14:0_16:0 | 0.325608236 | -0.376069944 |
| TG 15:0_16:0_16:0 | 0.285715331 | -0.351940384 |
| TG 12:0_14:0_16:1 | 0.371233844 | -0.457070258 |
| TG 14:0_15:0_16:0 | 0.145124077 | -0.319735352 |
| TG 12:0_14:0_16:0 | 0.249572277 | -0.460549741 |
| TG 16:0_18:0_22:0 | 0.178171859 | -0.39924716 |
| TG 16:0_16:1_18:1 | 0.216723617 | -0.4607191 |

Supplementary Table S2. Patient characteristics for RNA-seq

| Patient  case (#) | Sample type | Age (year) | Smoking  history | BMI (kg/m^2^) | Pre-operative PSA  (ng/ml) | Gleason score | pT  stage | N  stage |
| --- | --- | --- | --- | --- | --- | --- | --- | --- |
| #1 | prostate cancer | 72 | No | 22.2 | 29.82 | 8 | T3 | N0 |
| #2 | prostate cancer | 73 | No | 23.9 | 34.447 | 9 | T2 | N0 |
| #3 | prostate cancer | 66 | No | NA | 11.854 | 7 | T2 | N0 |
| #4 | prostate cancer | 74 | No | 25.4 | 31.072 | 8 | T3a | N0 |
| #5 | prostate cancer | 67 | No | 20.8 | 9.189 | 7 | T2 | NA |
| #6 | prostate cancer | 74 | No | 25.9 | 20.985 | 8 | T3 | N0 |
| #7 | prostate cancer | 57 | No | 22.9 | 15.193 | 7 | T2 | N0 |
| #8 | prostate cancer | 72 | No | 23.9 | 202.472 | 9 | T3b | N1 |
| #9 | prostate cancer | 67 | No | 20.4 | 153.544 | 9 | T3b | N0 |
| #10 | prostate cancer | 70 | No | 20.2 | 37.539 | 9 | T3b | N1 |
| #11 | prostate cancer | 66 | No | NA | 11.854 | 7 | T2 | N0 |
| #12 | prostate cancer | 65 | Yes | 23.4 | 11.6 | 7 | T2 | N0 |
| #13 | prostate cancer | 72 | No | 29.1 | 13.48 | 7 | T2 | N0 |
| #14 | prostate cancer | 66 | No | 24.5 | 50.414 | 8 | T3b | N0 |
| #15 | prostate cancer | 61 | No | 21.4 | 40 | 8 | T3a | N0 |
| #16 | prostate cancer | 72 | No | 21.8 | 13.666 | 7 | T2 | N0 |
| #17 | prostate cancer | 73 | No | NA | 17.756 | 7 | T2 | NA |
| #18 | prostate cancer | 62 | Yes | 27.8 | 15.644 | 7 | T2 | N0 |
| #19 | Peritumoral | 79 | No | 21.3 | 25.821 | 7 | T2 | N0 |
| #20 | Peritumoral | 78 | No | 25.8 | 27.141 | 7 | T2c | N0 |
| #21 | Peritumoral | 65 | No | 18.9 | 6.49 | 7 | T1a | N0 |
| #22 | Peritumoral | 78 | No | 21.4 | 30.2 | 8 | T2 | N0 |
| #10 | Peritumoral | 70 | No | 20.2 | 37.539 | 9 | T3b | N1 |
| #23 | Peritumoral | 68 | Yes | 25 | 12.983 | 10 | T3b | N1 |
| #24 | Peritumoral | 73 | No | 17.4 | 4.43 | 6 | T1a | N0 |
| #25 | Peritumoral | 84 | No | 24.913 | 79.9 | 7 | T2a | N0 |
| #26 | Peritumoral | 59 | Yes | 26.1 | 193.932 | 7 | T3b | N0 |

BMI: body mass index; PSA: prostate specific antigen; NA: not assessed; pT: pathological T

Supplementary Table S3. Patient characteristics for Triglyceride Content Test (Tumor tissues)

| Patient  case (#) | Age (year) | Smoking  history | BMI (kg/m^2^) | Pre-operative PSA  (ng/ml) | Gleason score | pT  stage | N  stage |
| --- | --- | --- | --- | --- | --- | --- | --- |
| #1 | 65 | No | 23.8 | 17.411 | 7 | T3 | N0 |
| #2 | 68 | Yes | 21.7 | 1.728 | 6 | T2 | N0 |
| #3 | 60 | No | 19.77 | 40.702 | 10 | T3a | N0 |
| #4 | 79 | No | 23.18 | 30.655 | 9 | T3b | N1 |
| #5 | 83 | Yes | 24.22 | 5.425 | 9 | T2 | N0 |
| #6 | 71 | No | 29.7 | 61.694 | 8 | T2 | N0 |
| #7 | 71 | No | 25.65 | 1.089 | 6 | T2 | NA |
| #8 | 75 | No | 24.49 | 13.685 | 9 | T2 | N0 |
| #9 | 65 | No | 22.98 | 39.952 | 6 | T2 | N0 |
| #10 | 69 | No | 23.38 | 5.79 | 8 | T2 | N0 |
| #11 | 59 | No | 25.05 | 8.895 | 6 | T2 | N0 |
| #12 | 60 | Yes | 23.83 | 0.220 | 8 | T3 | N1 |
| #13 | 64 | No | 23.03 | 26.843 | 7 | T2 | N0 |
| #14 | 62 | No | 25.6 | 154.867 | 7 | T3b | N0 |
| #15 | 65 | No | 23.3 | 17.262 | 7 | T2 | N0 |
| #16 | 67 | No | 23.44 | 55.953 | 9 | T3b | N1 |
| #17 | 61 | No | 29.38 | 71.288 | 9 | T2 | N0 |
| #18 | 68 | Yes | 25.15 | 3.601 | 7 | T2 | N0 |
| #19 | 74 | No | 25.15 | 2.043 | 6 | T1 | N0 |
| #20 | 78 | No | 21.4 | 11.99 | 6 | T2a | N0 |
| #21 | 72 | No | 22.1 | 16.804 | 7 | T4 | N0 |
| #22 | 70 | No | 21.97 | 11.8 | 6 | T2c | N0 |
| #23 | 81 | No | 21.63 | 17.427 | 9 | T2 | N1 |
| #24 | 62 | Yes | 24.15 | 102 | 9 | T3b | N1 |
| #25 | 70 | Yes | 22.77 | 7.441 | 7 | T2 | NA |
| #26 | 68 | No | 24.8 | 49.029 | 7 | T3b | N0 |
| #27 | 76 | No | 26.76 | 0.014 | 8 | T1 | N1 |
| #28 | 73 | No | 20.81 | 23.337 | 10 | T3b | N0 |
| #29 | 65 | No | 22.4 | 32.604 | 7 | T2 | NA |
| #30 | 70 | No | 22.83 | 95.21 | 9 | T3b | N0 |
| #31 | 62 | No | 23.62 | 6.116 | 7 | T3 | N0 |
| #32 | 75 | Yes | 23.12 | 52.893 | 7 | T3a | N0 |
| #33 | 68 | No | 24.33 | 15.91 | 9 | T3b | N1 |
| #34 | 71 | No | 27.1 | 98.1 | 8 | T2c | N0 |
| #35 | 68 | No | 19.1 | 0.008 | 7 | T2 | N0 |
| #36 | 74 | No | 29.64 | 14.473 | 7 | T2 | N0 |
| #37 | 65 | Yes | 27.2 | 4.06 | 7 | T2 | N0 |
| #38 | 79 | No | 22.9 | 3.396 | 8 | T2 | N0 |
| #39 | 72 | No | 27.5 | 13.946 | 10 | T4 | N0 |

BMI: body mass index; PSA: prostate specific antigen; NA: not assessed; pT: pathological T

Supplementary Table S4. Patient characteristics for Western blotting (paired samples)

| Patient  case (#) | Age (year) | Smoking  history | BMI (kg/m^2^) | Pre-operative PSA  (ng/ml) | Gleason score | pT  stage | N  stage |
| --- | --- | --- | --- | --- | --- | --- | --- |
| #1 | 70 | No | 20.415 | 57.512 | 7 | T2 | N0 |
| #2 | 57 | No | 22.8 | 12.8 | 7 | NA | NA |
| #3 | 71 | No | 21.9 | 326.746 | 9 | T2 | N0 |
| #4 | 65 | Yes | 23.4 | 11.6 | 7 | T2 | N0 |
| #5 | 60 | Yes | 20.8 | 16.353 | 7 | T2 | N0 |
| #6 | 64 | Yes | 18.4 | 31.6 | 9 | T2 | N0 |
| #7 | 82 | No | 24.4 | 9.597 | 9 | T2 | N0 |

BMI: body mass index; PSA: prostate specific antigen; NA: not assessed; pT: pathological T

Supplementary Table S5. Patient characteristics for IHC (paired samples)

| Patient  case (#) | Age (year) | Smoking  history | BMI (kg/m^2^) | Pre-operative PSA  (ng/ml) | Gleason score | pT  stage | N  stage |
| --- | --- | --- | --- | --- | --- | --- | --- |
| #1 | 68 | Yes | 26 | 1.589 | 7 | T2 | N0 |
| #2 | 64 | Yes | 18.4 | 31.6 | 9 | T2 | N0 |
| #3 | 71 | No | 21.9 | 9.63 | 8 | T3a | N0 |
| #4 | 59 | No | 25.2 | 17.848 | 6 | T3a | N0 |

BMI: body mass index; PSA: prostate specific antigen; NA: not assessed; pT: pathological T

Supplementary Table S6. Patient characteristics for RT-qPCR

| Patient  case (#) | Age (year) | Smoking  history | BMI (kg/m^2^) | Pre-operative PSA  (ng/ml) | Gleason score | pT  stage | N  stage |
| --- | --- | --- | --- | --- | --- | --- | --- |
| Paired samples | | | | | | | |
| #1 | 68 | No | 25.8 | 58 | 8 | T2 | N0 |
| #2 | 77 | No | 22.3 | 10.378 | 7 | T2a | N0 |
| #3 | 78 | No | 21.4 | 30.2 | 8 | T2 | N0 |
| #4 | 57 | Yes | 22.5 | 24.117 | 9 | T3b | N1 |
| #5 | 76 | Yes | 24.1 | 70.311 | 7 | T2c | N0 |
| #6 | 75 | No | 18 | 54.338 | 7 | T2 | N0 |
| #7 | 70 | No | 20.2 | 37.539 | 9 | T3b | N1 |
| #8 | 79 | No | 21.3 | 25.821 | 7 | T2 | N0 |
| #9 | 72 | No | 25.8 | 7.488 | 10 | T2 | N0 |
| Unpaired samples | | | | | | | |
| Tumor tissues | | | | | | | |
| #1 | 74 | No | 25.9 | 20.985 | 8 | T3 | N0 |
| #2 | 66 | No | NA | 11.854 | 7 | T2 | N0 |
| #3 | 70 | No | 25.7 | 14.532 | 7 | NA | NA |
| #4 | 61 | No | 21.4 | 40 | 8 | T3a | N0 |
| #5 | 67 | No | 20.4 | 153.544 | 9 | T3b | N0 |
| #6 | 72 | No | 29.1 | 13.48 | 7 | T2 | N0 |
| #7 | 70 | No | 23.9 | 1.82 | 6 | T2 | N0 |
| #8 | 69 | No | 26.298 | 9.223 | 8 | T2 | N0 |
| #9 | 67 | No | 20.4 | 40.839 | 7 | T2c | N0 |
| #10 | 70 | No | 24.5 | 50.414 | 8 | T3b | N0 |
| #11 | 57 | No | 22.9 | 15.193 | 7 | T3b | N1 |
| #12 | 76 | No | 27 | 19.024 | 9 | T3b | N1 |
| #13 | 57 | No | 23.4 | 12.731 | 7 | T3a | N0 |
| #14 | 72 | No | 22.2 | 29.82 | 8 | T3 | N0 |
| #15 | 65 | Yes | 23.4 | 11.6 | 7 | T2 | N1 |
| #16 | 63 | Yes | 25 | 48.549 | 7 | T3b | N1 |
| #17 | 72 | No | 21.8 | 13.666 | 7 | T2 | N0 |
| #18 | 72 | No | 22.1 | 16.804 | 7 | T4 | N0 |
| #19 | 70 | No | 21.97 | 11.8 | 6 | T2c | N0 |
| #20 | 81 | No | 21.63 | 17.427 | 9 | T2 | N1 |
| #21 | 62 | Yes | 24.15 | 102 | 9 | T3b | N1 |
| #22 | 70 | Yes | 22.77 | 7.441 | 7 | T2 | NA |
| #23 | 74 | No | 29.64 | 14.473 | 7 | T2 | N0 |
| #24 | 65 | Yes | 27.2 | 4.06 | 7 | T2 | N0 |
| #25 | 79 | No | 22.9 | 3.396 | 8 | T2 | N0 |
| #26 | 72 | No | 27.5 | 13.946 | 10 | T4 | N0 |
| #27 | 66 | Yes | 28.06 | 65.423 | 7 | T2 | N0 |
| Adjacent normal tissues | | | | | | | |
| #1 | 63 | No | 24.2 | 11.689 | 6 | T2b | N0 |
| #2 | 71 | No | 21.9 | 326.746 | 9 | T2 | N0 |
| #3 | 68 | Yes | 25 | 12.983 | 10 | T3b | N1 |
| #4 | 61 | Yes | 23.7 | 9.205 | 9 | T4 | N1 |
| #5 | 73 | No | 17.4 | 4.43 | 6 | T1a | N0 |
| #6 | 84 | No | 24.913 | 79.9 | 7 | T2a | N0 |
| #7 | 67 | Yes | 21.2 | 12.31 | 9 | T2 | N0 |
| #8 | 74 | No | 23.7 | 0.245 | 9 | T2 | N0 |
| #9 | 78 | No | 21.2 | 14.784 | 9 | T3b | N1 |
| #10 | 72 | Yes | 25.6 | 18.85 | 8 | T3a | N0 |
| #11 | 60 | Yes | 25.8 | 17.5 | 7 | T3a | N0 |
| #12 | 78 | No | 25.8 | 27.141 | 7 | T2c | N0 |

Table S7. Details of the cell lines

| Antibodies | SOURCE | Catalog No. |
| --- | --- | --- |
| PC3 | BeNa Culture Collection | 350783 |
| DU145 | BeNa Culture Collection | 100143 |
| RWPE-1 | MeisenCTCC | CTCC-003-0013 |
| LNcaP | BeNa Culture Collection | 337703 |
| 22RV1 | China Center for Type Culture Collection | GDC0288 |
| 293T | China Center for Type Culture Collection | GDC0187 |

Table S8. Details of the chemicals, critical commercial assays

| Chemicals, Critical commercial assays | SOURCE | Catalog No. |
| --- | --- | --- |
| MG132 | Medchemexpress | Cat#HY-13259 |
| RIPA buffer | Beyotime | Cat#P0013B |
| Triton X-100 | Solarbio | Cat#T8200 |
| Trypsin | Gibco | Cat#25200-072 |
| Polybrene | Yeasen | Cat#40804ES76 |
| DAPI | Invitrogen | Cat#D1306 |
| Penicillin-Streptomycin | Gibco | Cat#15140-122 |
| Fetal Bovine Serum (FBS) | Gibco | Cat#10099141 |
| PBS | Gibco | Cat#C10010500BT |
| DMEM high glucose | HYCLONE | Cat#SH30022.01 |
| RPMI 1640 | Gibco | Cat#C11875500BT |
| Lipofectamine 3000 Transfection Reagent | Invitrogen | Cat#L3000015 |
| Crystal Violet Staining Solution | Biosharp | Cat#BS051 |
| 4% paraformaldehyde | Biosharp | Cat#BL539A |
| DMSO | Sigma | Cat#D2650 |
| PhosSTOP™ | Roche | Cat#4906837001 |
| cOmplete™, EDTA-free Protease Inhibitor Cocktail | Roche | Cat#4693132001 |
| Cycloheximide | Medchemexpress | Cat#HY-12320 |
| Chloroquine | Medchemexpress | Cat#HY-17589A |
| WB primary antibody Diluent | Beyotime | Cat#P0023A |
| Prestained Protein Ladder | Vazyme | Cat#mp102-02 |
| EndoFree Maxi Plasmid Kit | TIANGEN | Cat. #DP117 |
| ReverTra Ace qPCR RT Kit | TOYOBO | Cat. #FSQ-101 |
| HiPure Total RNA Mini Kit | Magen | Cat. #R4111-03 |
| beaverBeads protein A/G immunoprecipitation Kit | BEAVER | Cat. # 22202-20 |
| CCK8 Kit | bimake | Cat. #B34304 |
| GSH and GSSG Assay Kit | Beyotime | Cat. #S0053 |
| Triglyceride assay kit | Nanjing Jiancheng | Cat. #A110-1-1 |
| BCA Protein Assay Kit | Beyotime | Cat. #P0012 |

Table S9. The siRNA and shRNA sequences used in this study.

| Gene | No. | siRNA sequence (5’-3’) |
| --- | --- | --- |
| GSTM4 | sh#1 | CCGGCGTTCCTTTCTCCTGTTTATTCTCGAGAATAAACAGGAGAAAGGAACGTTTTTG |
|  | sh#2 | CCGGGAAACTGAAGCCAGAATACTTCTCGAGAAGTATTCTGGCTTCAGTTTCTTTTTG |
|  | sh#3 | CCGGGCCCTGACTTTGAGAAACTGACTCGAGTCAGTTTCTCAAAGTCAGGGCTTTTTG |
| TRIM27 | si#1 | GGAAUCAUGGUCAUUCCAUTT |
|  | si#2 | GGCAGUGUCUUUGUGGUAUTT |
|  | si#3 | GCAGCUGUAUCACUCCUUATT |

Table S10. Primers used for the plasmid construction.

| Gene | Forward primer (5’-3’) | Reverse primer (5’-3’) |
| --- | --- | --- |
| Lentivirus GSTM4 overexpression | TCGGGTTTAAACGGATCCatgtccatgacactggg | GGGCCCTCTAGACTCGAGtcatctacagcagcccagaggcc |
| Lentivirus ACLY overexpression | TCGGGTTTAAACGGATCCatgtcggccaaggcaatttcagag | GGGCCCTCTAGACTCGAGttacatgctcatgtgttccggaagaacatatgaaat |
| Lentivirus GSTM4-MUT overexpression | cactggggGCCtgggacatccgcgg | ggatgtcccaGGCccccagtgtcatggacat |
| plasmid Flag-GSTM4 | TCGGGTTTAAACGGATCCatgtccatgacactggg | GGGCCCTCTAGACTCGAGtcatctacagcagcccagaggcc |
| plasmid HA-GSTM4 | TCGGGTTTAAACGGATCCatgtccatgacactggg | GGGCCCTCTAGACTCGAGtcatctacagcagcccagaggcc |
| plasmid Myc-GSTM4 | TCGGGTTTAAACGGATCCatgtccatgacactggg | GGGCCCTCTAGACTCGAGtcatctacagcagcccagaggcc |
| plasmid Flag-GSTM4（1-88） | TCGGGTTTAAACGGATCCatgtccatgacactggggTACtg | GGGCCCTCTAGACTCGAGttacccacacaggttgtgcttgc |
| plasmid Flag-GSTM4（89-218） | TCGGGTTTAAACGGATCCatggagacagaagaggagaagattcgtgtg | GGGCCCTCTAGACTCGAGttacttgttgccccagacagcc |
| plasmid GST-HA-GSTM4 | TCGGGTTTAAACGGATCCatgtccatgacactggg | GGGCCCTCTAGACTCGAGtcatctacagcagcccagaggcc |
| plasmid Flag-ACLY | TCGGGTTTAAACGGATCCatgtcggccaaggcaatttcagag | GGGCCCTCTAGACTCGAGttacatgctcatgtgttccggaagaacatatgaaat |
| plasmid HA-ACLY | TCGGGTTTAAACGGATCCatgtcggccaaggcaatttcagag | GGGCCCTCTAGACTCGAGttacatgctcatgtgttccggaagaacatatgaaat |
| plasmid HA-ACLY（1-440） | TCGGGTTTAAACGGATCCatgtcggccaaggcaatttcagag | GGGCCCTCTAGACTCGAGttagttgaggaggaagtttgcagtgtgggc |
| plasmid HA-ACLY（441-778） | TCGGGTTTAAACGGATCCatggccagcgggagcacatcgac | GGGCCCTCTAGACTCGAGttaagcctggttcttggctactgcagttt |
| plasmid HA-ACLY（779-1101） | TCGGGTTTAAACGGATCCatgttgaaggaagcaggagtgtttgtgcc | GGGCCCTCTAGACTCGAGttacatgctcatgtgttccggaagaacatatgaaat |
| plasmid GST-HA-ACLY | TCGGGTTTAAACGGATCCatgtcggccaaggcaatttcagag | GGGCCCTCTAGACTCGAGttacatgctcatgtgttccggaagaacatatgaaat |
| plasmid Flag-TRIM27 | TCGGGTTTAAACGGATCCatggcctccgggagtgtgg | GGGCCCTCTAGACTCGAGtcaaggggaggtctccatggaatgac |
| plasmid HA-TRIM27 | TCGGGTTTAAACGGATCCatggcctccgggagtgtgg | GGGCCCTCTAGACTCGAGtcaaggggaggtctccatggaatgac |
| plasmid Flag-TRIM27（1-127） | TCGGGTTTAAACGGATCCatggcctccgggagtgtgg | GGGCCCTCTAGACTCGAGtcagtggccgcggtgctcgcgggag |
| plasmid Flag-TRIM27（128-513） | TCGGGTTTAAACGGATCCatgagcgtgctgccgctcgaggaggcg | GGGCCCTCTAGACTCGAGtcaaggggaggtctccatggaatgac |
| plasmid HA-PDLIM2 | TCGGGTTTAAACGGATCCatggcgttgacggtggatgtggccg | GGGCCCTCTAGACTCGAGtcaggcccgagagctgagggtggcagg |
| plasmid HA-PRPF19 | TCGGGTTTAAACGGATCCatgtccctaatctgctccatctctaacg | GGGCCCTCTAGACTCGAGctacaggctgtagaacttgaggcttc |
| plasmid HA-PWP2 | TCGGGTTTAAACGGATCCatgaagttcgcttaccggttttcaaatttg | GGGCCCTCTAGACTCGAGctaggccagcatctcttcttctgagtc |
| plasmid HA-CORO2A | TCGGGTTTAAACGGATCCatgtcatggcacccccagtac | GGGCCCTCTAGACTCGAGtcagagctgctctgagcccatcc |
| plasmid HA-UPF1 | TCGGGTTTAAACGGATCCatgagcgtggaggcgtacg | GGGCCCTCTAGACTCGAGttaatactgggacagccccgtcacc |
| plasmid HA-WDR33 | TCGGGTTTAAACGGATCCatggctacagaaattggttctcctcctcg | GGGCCCTCTAGACTCGAGctaccgaccccttccaccacc |

Table S11. Details of the antibodies used in this study.

| Antibodies | SOURCE | Catalog No. |
| --- | --- | --- |
| anti-GSTM4, dil:1:1,000 | proteintech | Cat. #16766-1-AP |
| anti-ACLY, dil:1:2,000 | Abclonal | Cat. #A3719 |
| anti-TRIM27, dil:1:1,000 | Cell Signaling Technology | Cat. #15099 |
| anti-PCNA, dil:1:1,000 | proteintech | Cat. #10205-2-AP |
| anti-CyclinD1, dil:1:1,000 | Cell Signaling Technology | Cat. #2978S |
| anti-MMP9, dil:1:1,000 | Cell Signaling Technology | Cat. #13667S |
| anti-E-cadherin, dil:1:500 | Cell Signaling Technology | Cat. #3195 |
| anti-N-cadherin, dil:1:500 | Cell Signaling Technology | Cat. #13116 |
| anti-FASN, dil:1:1,000 | Abclonal | Cat. #A19050 |
| anti-ACC1, dil:1:1,000 | Abcam | Cat. #ab45174 |
| anti-HMGCR, dil:1:1,000 | Abcam | Cat. #ab174830 |
| anti-SQLE, dil:1:1,000 | Proteintech | Cat. #12544-1-AP |
| anti-β-Actin, dil:1:1,000 | Abclonal | Cat. #AC026 |
| anti-Mouse-Flag, dil:1:1,000 | Sigma | Cat. #F1804 |
| anti-Rabbit-DDDDK, dil:1:1,000 | Abclonal | Cat. #AE092 |
| anti-Mouse-HA, dil:1:1,000 | origene | Cat. #TA180128 |
| anti-Rabbit-HA, dil:1:1,000 | Abclonal | Cat. #AE105 |
| anti-Mouse-Myc, dil:1:1,000 | Cell Signaling Technology | Cat. #2276 |
| Anti-Mouse-IgG (H+L)-HRP, dil:1:10,000 | Jackson ImmunoResearch Inc | Cat. #115-005-003 |
| Anti-Rabbit-IgG (H+L)-HRP, dil:1:10,000 | Jackson ImmunoResearch Inc | Cat. #111-005-003 |
| anti-GST Tag, dil:1:1,000 | Proteintech | Cat. #10000-0-AP |

Table S12. Primers used for the qRT-PCR assay.

| Gene | Forward primer (5’-3’) | Reverse primer (5’-3’) |
| --- | --- | --- |
| β-actin | CATGTACGTTGCTATCCAGGC | CTCCTTAATGTCACGCACGAT |
| GSTM4 | CAACTGCTTGGACGCCTTTC | AGAGGTTTTGGGAGGAAGCG |
| ACLY | GAAGCTGACCTTGCTGAACC | CTGCCTCCAATGATGAGGAT |

qRT-PCR: quantitative reverse transcription PCR.
